# Ferritins as natural and artificial nanozymes for theranostics

**DOI:** 10.7150/thno.39827

**Published:** 2020-01-01

**Authors:** Bing Jiang, Long Fang, Kongming Wu, Xiyun Yan, Kelong Fan

**Affiliations:** 1 Joint Laboratory of Nanozymes in Zhengzhou University, Academy of Medical Sciences, Zhengzhou University, 40 Daxue Road, Zhengzhou 450052, China.; 2 CAS Engineering Laboratory for Nanozyme, Key Laboratory of Protein and Peptide Pharmaceutical, Institute of Biophysics, Chinese Academy of Sciences, 15 Datun Road, Beijing 100101, China.; 3 Savaid Medical School, University of Chinese Academy of Sciences, Beijing 100049, China.; 4 Department of Medical Oncology, The Affiliated Cancer Hospital of Zhengzhou University & Henan Cancer Hospital, Zhengzhou, 450008, China.

**Keywords:** Ferritins, nanozymes, theranostics

## Abstract

Nanozymes are a class of nanomaterials with intrinsic enzyme-like characteristics which overcome the limitations of natural enzymes such as high cost, low stability and difficulty to large scale preparation. Nanozymes combine the advantages of chemical catalysts and natural enzymes together, and have exhibited great potential in biomedical applications. However, the size controllable synthesis and targeting modifications of nanozymes are still challenging. Here, we introduce ferritin nanozymes to solve these problems. Ferritins are natural nanozymes which exhibit intrinsic enzyme-like activities (*e.g.* ferroxidase, peroxidase). In addition, by biomimetically synthesizing nanozymes inside the ferritin protein shells, artificial ferritin nanozymes are introduced, which possess the advantages of versatile self-assembly ferritin nanocage and enzymatic activity of nanozymes. Ferritin nanozymes provide a new horizon for the development of nanozyme in disease targeted theranostics research. The emergence of ferritin nanozyme also inspires us to learn from the natural nanostructures to optimize or rationally design nanozymes. In this review, the intrinsic enzyme-like activities of ferritin and bioengineered synthesis of ferritin nanozyme were summarized. After that, the applications of ferritin nanozymes were covered. Finally, the advantages, challenges and future research directions of advanced ferritin nanozymes for biomedical research were discussed.

## 1. Introduction

**Figure 1:**
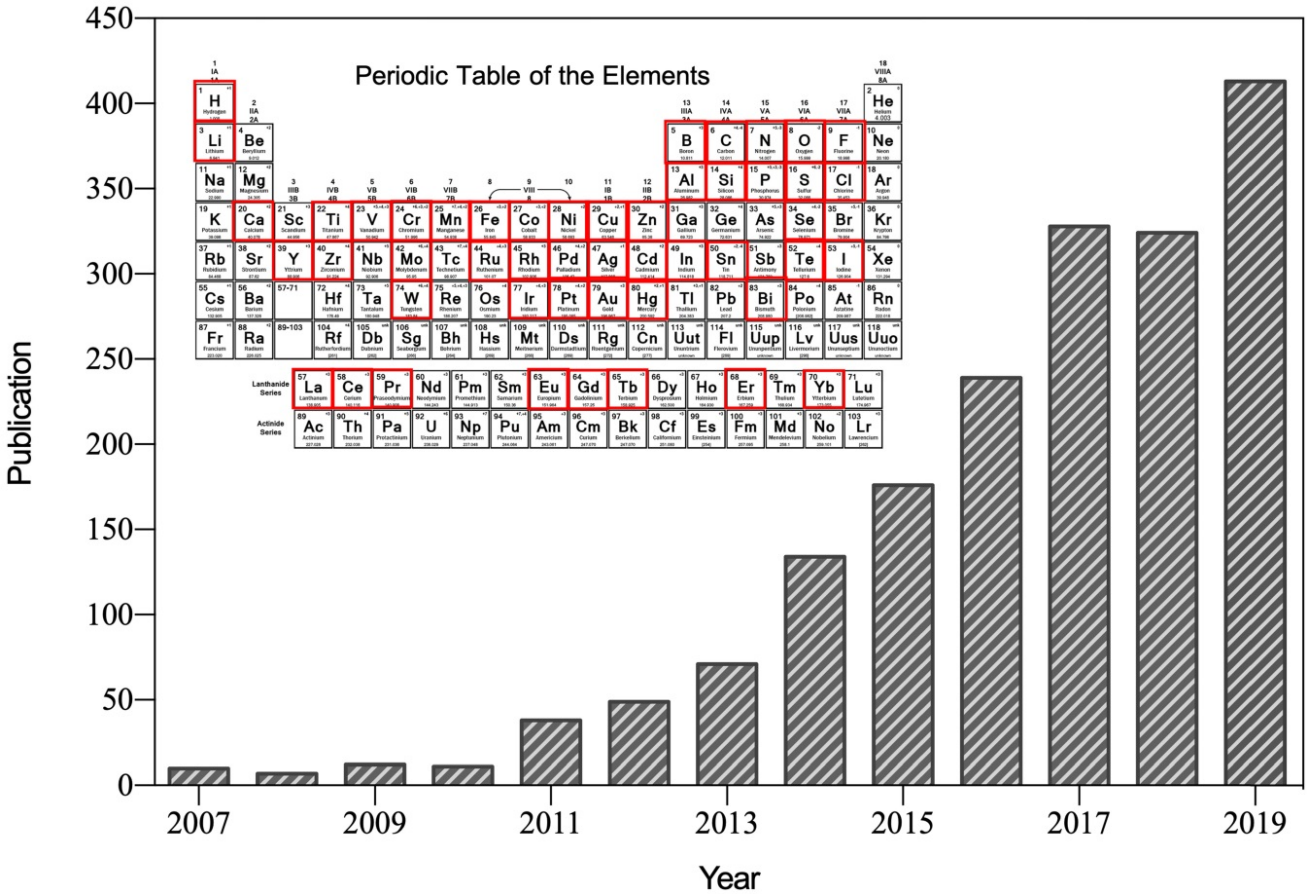
Number of published papers on nanozyme research included by the Web of Science by the end of August 2019. Until now, more than 49 elements have been employed to synthesize nanozyme.

After the first reported suggesting that ferromagnetic (Fe_3_O_4_) nanoparticles possess intrinsic peroxidase-like activity in 2007 1, nanozyme was specifically referred to the nanomaterials with intrinsic enzyme-like characteristics (Figure 1). Nanozymes can catalyze the substrates of natural enzymes under mild conditions, and possess similar catalytic capability and reaction kinetics as natural enzymes 2. Compared with natural enzymes, nanozymes possess higher catalytic stability, more economic and ease of large-scale preparation 2, 3. In addition, nanozymes possess inherent nanomaterial properties, such as light, magnetism, heat, and so on, which are able to be designed to suitable for specific biological microenvironment and external stimulations 4. The superiorities of nanozymes over natural enzymes attracted many scientists devoting to the research filed of nanozyme. Emerging studies have since then revealed that various nanomaterials (such as metal and metallic oxide nanoparticles, carbon nanoparticles, metal-organic frameworks (MOFs), and so on) exhibit enzyme-like activities 5. Nanozyme is regarded as a new material and next-generation artificial enzyme, and the concept of nanozyme is well accepted now. More than 350 research laboratories from 30 countries have been working on nanozymes, and more than 540 types of nanozymes synthesized from 49 elements have been reported (Figure 1). By bridging the nanotechnology and biology, nanozymes have emerged as novel promising agents for disease theranostic strategies 6.

However, size-controllable synthesis and targeting modifications of nanozymes are still challenging in the theranostic applications. Typically, the enzyme-like activity of nanozymes is size-dependent. The smaller size is, the larger specific surface area of nanozymes is, and the higher relative enzyme-like activity of nanozymes is 1. Thus, size-controllable synthesis of nanozymes is important for stabilizing the theranostic effect of nanozymes. Moreover, when nanozymes are used for disease-targeted theranostics, nanozymes are typically chemically modified with targeting motifs, such as antibodies 7. This chemical coupling procedure is practicable under the premise of that nanozymes possess amidogen, carboxyl or sulfhydryl on its surface. However, some nanozymes (such as Prussian blue) are not suitable for chemical group modification 8. In addition, these chemical conjugates require complicated multi-step chemical reactions and expensive reagents 9. Thus, targeting modifications of nanozymes need new strategies.

The emergence of ferritin nanozymes provides a promising strategy to solve these challenges. Ferritin, an iron storage protein nanocage, is a natural nanomaterial composed of organic and inorganic components, which possesses constant nanosized cavity and intrinsic enzyme-like activities for biomineralization. Therefore, ferritin can be used as nanozyme carrier or reactor for size-controllable synthesis of nanozymes. Moreover, ferritin nanocages possess both passive and active tumor targeting abilities, which have been widely used for tumor targeted theranostics 10, 11. In recent works, we identified that ferritin can target tumor tissues by recognizing transferrin receptor 1 (TfR1) which is highly expressed in tumors 9, 12-15. Taking the advantage of biomineralization process of ferritin, we synthesized different types of metallic oxide nanozymes in the cavity of ferritin with a confined size 12, 16, 17. Thus, ferritin nanozyme can be used for tumor targeted theranostics. Through genetic or chemical approaches, we modified ferritin nanocages with targeting moieties to endow different targeting abilities of ferritin nanozymes 16. Ferritin nanozymes possess the advantages of uniform size, modifiable targeting ability, good compatibility, thus ferritin nanozyme is a superior strategy for size-controllable synthesis and targeting modifications of nanozymes.

Here, in this review, we systematically introduced the concept and development of ferritin nanozyme, as well as the recent research progress of ferritin nanozyme. In detail, we summarized the natural enzyme-like activities of ferritin, ferritin as a nanoreactor to fabricate various nanozymes with a confined size and endow nanozymes with different targeting abilities, and the disease-targeted biomedical applications of ferritin nanozymes. Finally, the challenges and perspectives are discussed for future exploration in this filed. We expect to find the rules for the synthesis of different kinds of nanozymes inside the ferritin nanocage through learning the way of biomineralization process of ferritin. Moreover, we expect to design new kinds of ferritin nanozymes to better adapt the complicated biological microenvironment and intelligent response to specific stimulations.

## 2. Ferritins as natural nanozymes

### 2.1 The structure of ferritin

**Figure 2:**
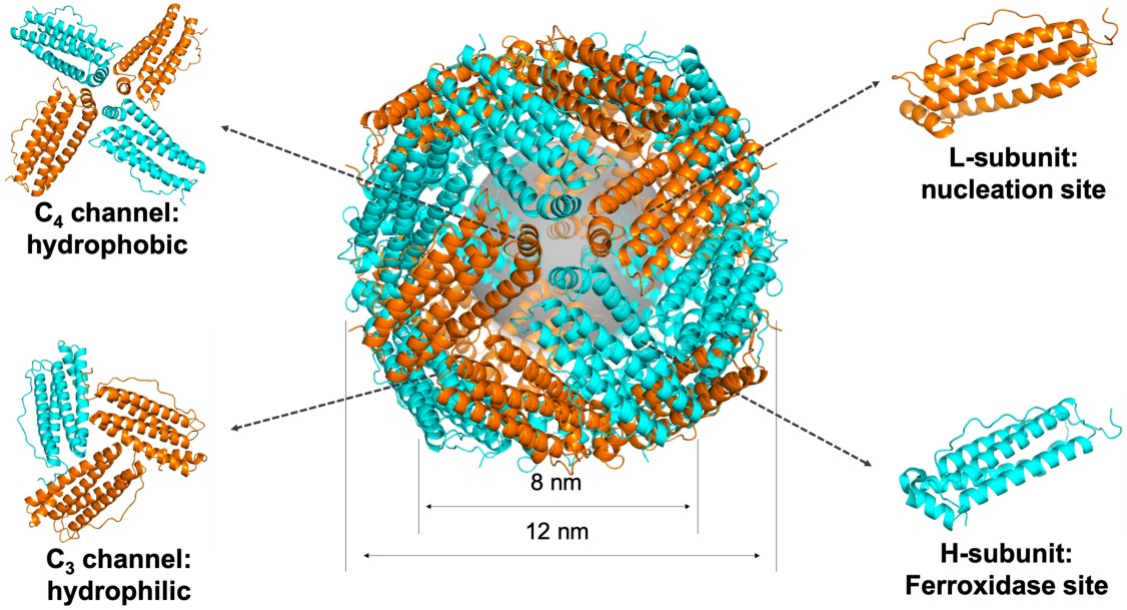
The structure of ferritin. Ferritin is a 24-subunit oligomer with a combination of H-subunit (ferroxidase site) and L-subunit (nucleation site) that self-assemble into a spherical symmetrical protein shell, with inner and outer diameter of 8 and 12 nm, respectively. Eight hydrophilic channels and six hydrophobic channels are formed on the protein shell. Ferrihydrite core is synthesized in the cavity (PDB entry 5N27).

Ferritin is a spherical iron storage protein which is widely existed in organisms. In 1937, ferritin was first isolated from horse spleen 18, since then, ferritin has been found in humans, animals, plants, fungi and bacteria 19. The amino acid sequence of ferritin from different species varies a lot, while the protein structure keeps consistent 20. Naturally, 24 ferritin subunits self-assembled into a spherical protein cage, with inner and outer diameter of 8 and 12 nm, respectively (Figure 2). Ferritin protein cage encapsulates up to 4500 Fe^3+^ atoms into its cavity in the form of iron core. The iron core of ferritin consists of a ferric-oxyhydroxide-phosphate complex 21. Typically, the phosphate contained in the complex is dissolved when it is isolated out of the protein cage by heat or denaturants treatment, and the remaining product is ferrihydrite, 5Fe_2_O_3_·9H_2_O 22. After removing the mineral core located in the cavity, we can get the ferritin protein shell, called apoferritin.

Each subunit of ferritin consists of a bundle of four long helixes, a short helix, and a long loop 20. The N-terminal of each subunit exposes on the outer surface of ferritin nanocage, while the C-terminal folds into the inner cavity 19. The ferritin nanocage possesses six C_4_ channels and eight C_3_ channels, which form the symmetrical structure of ferritin protein cage 22 (Figure 2). The C_3_ channels show hydrophilic property, allow metal ions, like Fe^2+^ ion, and H_2_O molecule to enter the cavity. The C_4_ channels show hydrophobic property, allowing O_2_ or small organic molecules to enter the cavity 20.

Ferritin subunits self-assembled into a spherical shell through the hydrophobic interaction 20. There are many hydrogen bonds and salt bridges within the subunits 23, so that ferritins stay stable even at high temperature environment and tolerate many denaturants, such as guanidine hydrochloride and urea. Under the condition of high concentration of guanidine hydrochloride (6M) or urea (8M), ferritin protein shell is disassembled. After removing these denaturants, ferritin subunits will re-assemble into a protein shell 24. Under the environment of extreme acid (pH 2-3) or extreme alkaline (pH 10-12), ferritin protein shell disassembles. When it recovers to the physiological environment, ferritin subunits will also re-assemble into a protein shell 25. These characteristics of ferritin make it a useful platform as nanocarrier or nanoreactor for biomedical applications.

Mammalian ferritins, like human ferritin, typically consist of two different types of subunits, H-subunit and L-subunit (Figure 2). The molecular weights of H-subunit and L-subunit are 21 kDa and 19 kDa, respectively. There are evidences that H-subunits and L-subunits have cooperative roles in the iron-storage mechanism of human ferritin 26.

### 2.2 Ferritin protein shell shows ferroxidase-like activity

In fact, ferritin as a natural nanomaterial is also a kind of nanozyme, which possesses intrinsic ferroxidase-like activity. In 1973, it was firstly found that horse spleen apoferritin (HSAF) possesses catalytic activity, which catalyzes the oxidation of Fe^2+^ to Fe^3+^ with molecular O_2_ as electron acceptor where other proteins have no such effect 27. Soon afterwards, it was reported that ferritin H-subunit contains a ferroxidase site 28, which catalyzes the oxidation of ferrous ion into ferric iron rapidly, whereas the L-subunit doesn't have. L-subunit facilitates the iron oxidation at ferroxidase site and promotes oxidation on the mineral surface once the iron binding capacity of the ferroxidase site was exceeded, thus L-subunit facilitates the nucleation of ferrihydrite 29. Thereby, in mammal ferritin, H-subunit rich ferritins exhibit fast iron incorporation rate, which can rapid catalyze the oxidation of toxic Fe^2+^ into nontoxic Fe^3+^, are suitable for high metabolic activity tissues, such as heart and brain. Whereas L-subunit rich ferritins exhibit lower iron incorporation rate, but store more iron. This characteristic is found in iron-rich tissues, such as liver and spleen.

**Figure 3:**
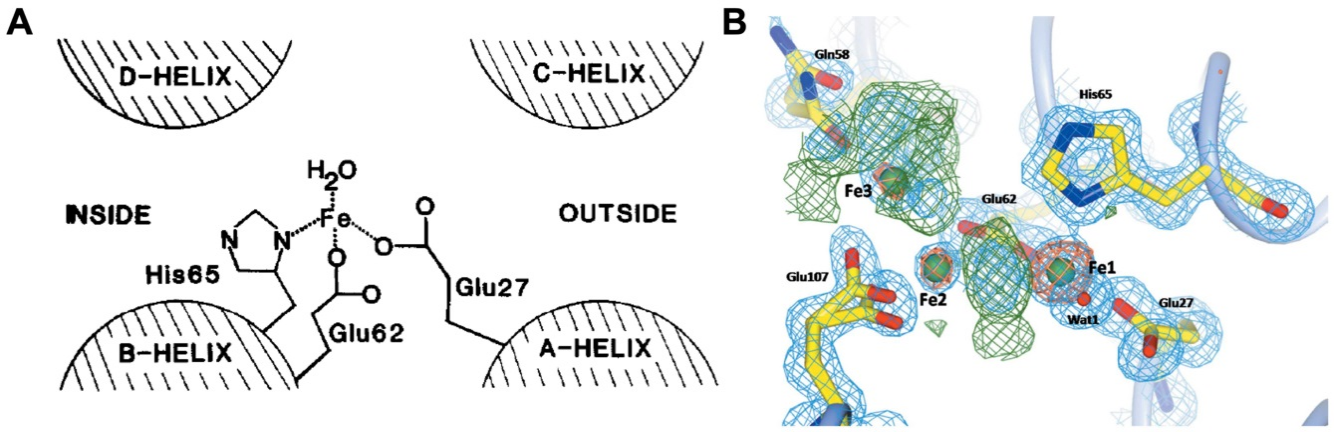
The ferroxidase site of ferritin H-subunit. (A) The proposed ferroxidase center in recombinant H-subunit ferritin. Reprinted with permission from 30. Copyright 2015 Federation of European Biochemical Societies. (B) Electron density in the Human ferritin H-subunit oxidoreductase site after 1 min of free diffusion of iron into the crystals. Reprinted with permission from 32. Copyright 2015 International Union of Crystallography.

Owing to the high ferroxidase-like activity of ferritin H-subunit, the iron-core formation within ferritin is much faster than the formation of ferric oxyhydroxide under protein-free conditions 20. The ferroxidase site of human H-subunit mainly consists of three amino acid residues (Glu 27, Glu 62, His 65) 30 (Figure 3 A). These three residues are conserved in most known H-subunit sequences from different species, but are absent in L-subunit sequences 19. It is reported that ferritin H-subunit lost its ferroxidase-like activity after mutation of two amino acid residues, E62K and H65G (using amino acids of L-subunits to replace H-subunits on 62 and 65) 30. Later on, in 1996, Harrison and Arosio presented a di-iron ferroxidase center model, which indicated that there were two iron bind sites (Fe_A_ and Fe_B_) in the ferroxidase center 20, and they showed that the inner surface amino acid residues Glu 62, Glu 107, Tyr 34, Gln 141 formed the second iron bind site (Fe_B_) in the ferroxidase center 31. Furthermore, it was reported that Gln 58 may also possess iron bind ability 32 (Figure 3B). Moreover, the inner surface Glu residues of human ferritin H-subunit located nearby the ferroxidase site contribute to the deposition of iron core, which is supported by the results that mutation of both ferroxidase site and inner surface Glu residues in human ferritin H-subunit resulted in completely disappearance of iron oxidation and nucleation 33.

Ferritin isolated from microorganisms, such as bacterial ferritins, is similar to that of human ferritin H-subunit, with subunit molecular weight around 20 kDa 19. These ferritins also possess ferroxidase site like human ferritin H-subunit, which can catalyze the oxidation of Fe^2+^ to Fe^3+^ and store iron in the cavity of ferritin 34.

The possible procedure of iron-core formation inside the ferritin nanocage contains 4 steps 20, 22, 28, 29, 31, 35, 36.

1. Fe^2+^ ion traversing the ferritin protein shell. Due to the hydrophilic property of C_3_ channels on the surface of ferritin nanocage, Fe^2+^ ions can freely enter the cavity of ferritin nanocage through C_3_ channels and then move to the ferroxidase site.

2. Fe^2+^ ion oxidation. After reaching to the ferroxidase site of ferritin H-subunits, Fe^2+^ ions bind to the ferroxidase catalytic site, then oxidation occurred in the presence of O_2_ resulting in the generation of H_2_O_2_ 36.

Protein + 2 Fe^2+^ + O_2_ + 3 H_2_O → Protein-[Fe_2_O(OH)_2_]^2+^ + H_2_O_2_ + 2 H^+^. (1)

3. The oxidized Fe^3+^ atoms move to the nucleation site of the cavity, hydrolyze and form the mineral core^35^.

Protein-[Fe_2_O(OH)_2_]^2+^ + H_2_O → Protein + 2FeOOH(core) + 2 H^+^. (2)

4. Once the iron core nuclei have reached 100 or more Fe^3+^ atoms, Fe^2+^ ions directly oxide and deposit on the surface of the mineral core in the presence of H_2_O_2_ 35. So that, along with the consumption of Fe^2+^ ions and H_2_O_2_, the size of the mineral core increases constantly. Finally, a stable ferrihydrite mineral core is formed under the confined the size of the ferritin cavity.

2 Fe^2+^ + H_2_O_2_ + 2 H_2_O → 2 FeOOH_(core)_ + 4 H^+^. (3)

### 2.3 Natural isolated ferritins show other enzyme-like activities

**Figure 4:**
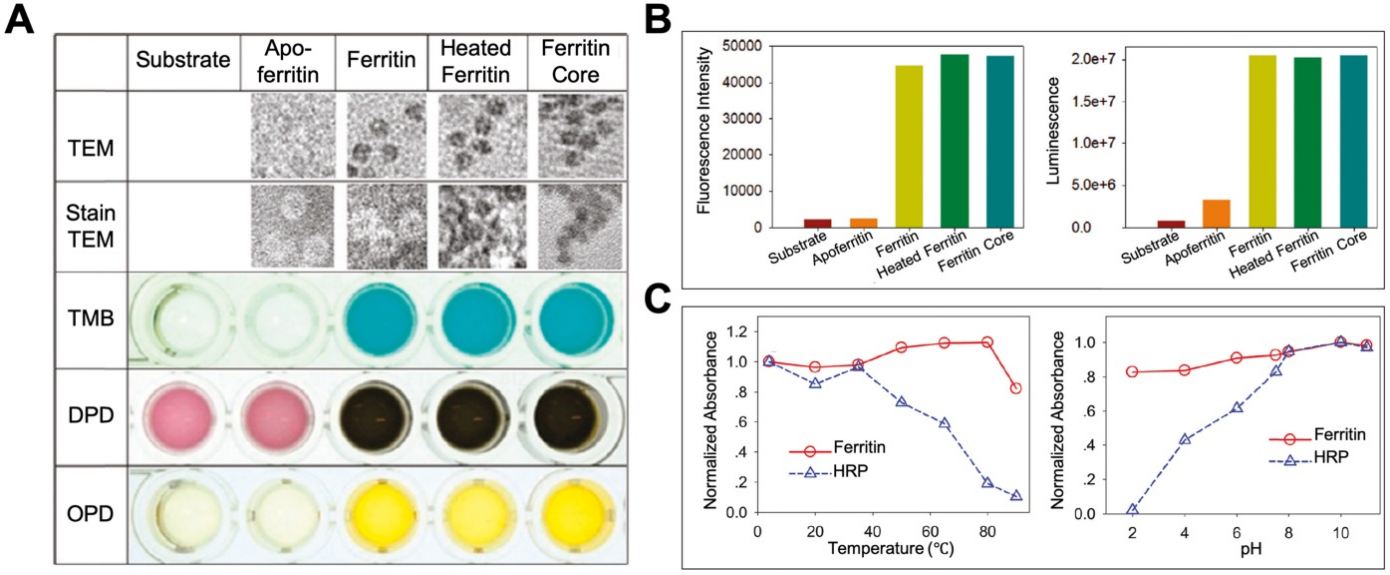
The peroxidase activity of ferritin. (A) TEM images of ferritin samples and the analysis of its peroxidase-like activity using TMB, DPD, and OPD as substrates at 50 ℃. (B) Analysis of peroxidase-like activity of ferritin using p-HPPA (left) and luminol (right) as substrates. (C) The comparison of thermal stability (left) and pH tolerance (right) of peroxidase activity between ferritin and HRP. Reprinted with permission from 41. Copyright 2011 American Chemical Society.

By exerting its ferroxidase-like activity, ferritin plays an important role in antioxidation and detoxification metabolisms 31. Some studies also showed that except for the iron binding and ferroxidase-like activity, ferritin also showed peroxidase-like activity 37-41, which may endow ferritin more functions in modulating the redox metabolism. In 1997, Arapova, G. S. *et al* first found that horse spleen ferritin possesses intrinsic peroxidase activity, which catalyzes the oxidation of 3,3',5,5'-tetramethylbenzidine (TMB), ortho-tolidine, and ortho-phenylenediamine (PDA) in the presence of H_2_O_2_ at 20 ℃ 37. In 2011, Tang *et al* also confirmed this phenomenon by proving that horse spleen ferritin catalyzes the oxidation of TMB, o-phenylenediamine (OPD), N,N-dimethyl-p-phenylenediamine (DPD) (Figure 4 A), p-hydroxyphenylpropionic acid (p-HPPA), and luminol (Figure 4 B) in the presence of H_2_O_2_ at 50 ℃ for 15 minutes 41. They found that horse spleen ferritin still exhibits high peroxidase activity even at high temperature (over 80 ℃) or extreme acid environment (pH 2.0). The thermal stability and pH tolerance of the peroxidase activity of horse spleen ferritin are superior to those of horseradish peroxidase (HRP) (Figure 4 C). In 2012, Violetta Borelli *et al* reported that ferruginous bodies, of which human lung ferritin is the main proteinaceous component, possess peroxidase-like activity 40. These studies indicate that natural ferritins isolated from different spices exhibit intrinsic peroxidase activity 37-41.

Interestingly, Arapova, G. S. *et al* showed that the peroxidase activity of ferritin was decreased after thermal inactivation and detergents treatment, which indicated that the peroxidase activity of ferritin was derived from ferritin protein shell 37. Whereas, Tang *et al* showed that the peroxidase-like activity of horse spleen ferritin was derived from its ferric nanocore but not the ferritin protein shell 41. Which component of ferritin, iron core or protein shell, is responsible for the peroxidase activity needs further clarification. These catalytic activities of ferritin, derived from whether protein shell or iron core, inspired us to utilize ferritin as a nanoreactor or nanocarrier for biomedical applications by exerting its catalytic activities.

**Figure 5:**
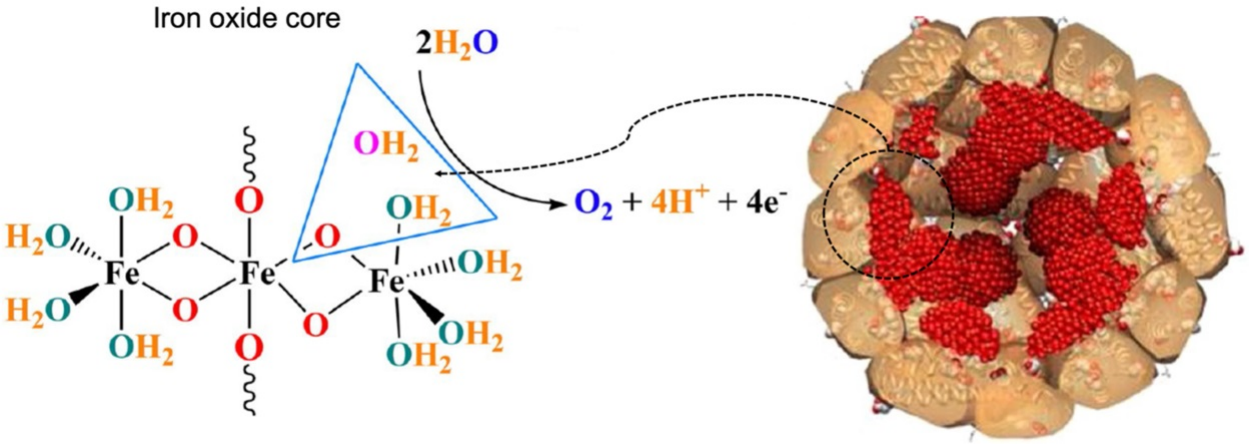
Water oxidation catalyzed by the iron oxide core of horse spleen ferritin. Reprinted with permission from 42. Copyright 2019 Springer Nature.

Apart from ferroxidase- and peroxidase-like activities, ferritin also exhibits other types of catalytic activities. Recently, it was reported that horse spleen ferritin was an iron-based catalyst which catalyzed water oxidation and released molecular oxygen under alkaline environment (pH 11.0) 42 (Figure 5). The authors declared that iron oxide core of horse spleen ferritin responded for this catalytic activity. When iron inside the horse spleen ferritin nanocage was consumed by bacteria, its water oxidation activity was reduced.

Horse spleen ferritin was also reported being a photocatalyst which catalyzes the synthesis of gold nanoparticle on the surface of horse spleen ferritin. The authors regarded that the outer surface of horse spleen ferritin possessed a putative nucleation site which binds Au^3+^ ions 43. The ferrihydrite mineral core of horse spleen ferritin possesses semi-conductor capacity 43. Photochemical excitation of the ferrihydrite core transfers electrons across the horse spleen ferritin protein shell to photoreduce AuCl^4-^ to form gold nanoparticles in the putative surface nucleation site of horse spleen ferritin 43.

## 3. Ferritins as artificial nanozymes

### 3.1 Ferritin as a template to synthesis inorganic metal-based nanomaterials

Due to the unique fixed nanosized cavity structure and eight negatively charged hydrophilic channels on the protein shell, Fe^2+^ and other positively charged metal ions can enter the cavity of ferritin protein shell through hydrophilic channels freely. Then these metal ions can be catalyzed to form metallic oxide core at the ferroxidase site of the cavity. Taking advantages of these characteristics of ferritin, it would be an efficient approach to synthesize inorganic metal-based nanomaterials in a size-controllable manner by using ferritin as a template (Table 1).

A series of inorganic metallic oxide nanoparticles have been reported to be synthesized in the 8 nm cavity of ferritin by using ferritin as a nanoreactor (Table 1), including Fe_3_O_4_ 12, 44, 45, MnOOH 46, 47, In_2_O_3_ 48, TiO_2_ 49, EuOOH 49, Co_3_O_4_ 16, 50 , Ni(OH)_3_ 51, Cr(OH)_3_ 51, FeS 52, CdS 53, PbS 54, ZnSe 55, FePO_4_ 56 nanoparticles. The additional oxidizing agents, such as H_2_O_2_ accelerates the oxidation of metal ions, and additional NaOH promotes the mineralization and nucleation.

Moreover, some metal ions can bind to the inner surface of ferritin nanocage through the electrostatic interaction with hydrophilic channels. In the presence of some reducing agents, such as ascorbic acid, these metal ions were reduced into metal atoms, and then aggregated into metal nanoparticles in the cavity of ferritin. Through this way, some metal or metal alloy nanoparticles have been synthesized in ferritin nanocages (Table 1), such as Au 57, Ag 58, Cu 58, Pd 59, Co 58, 60, Ni 58, 60, Pt 61, CoPt 62, AuPd 63.

When metal ions bind to the cavity of ferritin nanocages and phosphates are added to the solution, metal phosphates nanoparticles can be synthesized in the cavity of ferritin nanocages through the phosphitylation process. By this way, silver phosphate nanoparticles were synthesized in the cavity of HSAF nanocages with a controlled size 64.

Compared with other methods for the synthesis of nanoparticles, using ferritin as a nanoreactor to synthesize nanoparticles in its cavity has several advantages 48, 52. (1) Uniform size. Owing to the confined size of inner cavity and the accurate catalysis of protein shell, the biomineralization of nanomaterials inside ferritin was controllable and efficient. The size of the synthesized nanoparticles was confined under 8 nm and uniform. (2) Monodisperse and good biocompatibility. The synthesized nanoparticles were nanocaged by ferritin protein shell, thus the monodisperse state, good biocompatibility and low biotoxicity of ferritin protein shell shall not be affected. (3) The nanoparticles were synthesized inside the ferritin nanocage under near room temperature and mild environment, which differentiates from the normal chemical synthesis typically needing high temperature, high pressure and extreme acid or alkaline environment. (4) Ease of surface functional modifications. Through genetic or chemical approaches, functional motifs, such as targeting molecules, therapeutic drugs, can be easily modified onto ferritin nanocages. Thus, we can endow nanoparticles synthesized inside the ferritin nanocages with specific functions by surface functional modifications of ferritin protein shell.

### 3.2 Ferritin as a nanoreactor to synthesize nanozymes

**Figure 6:**
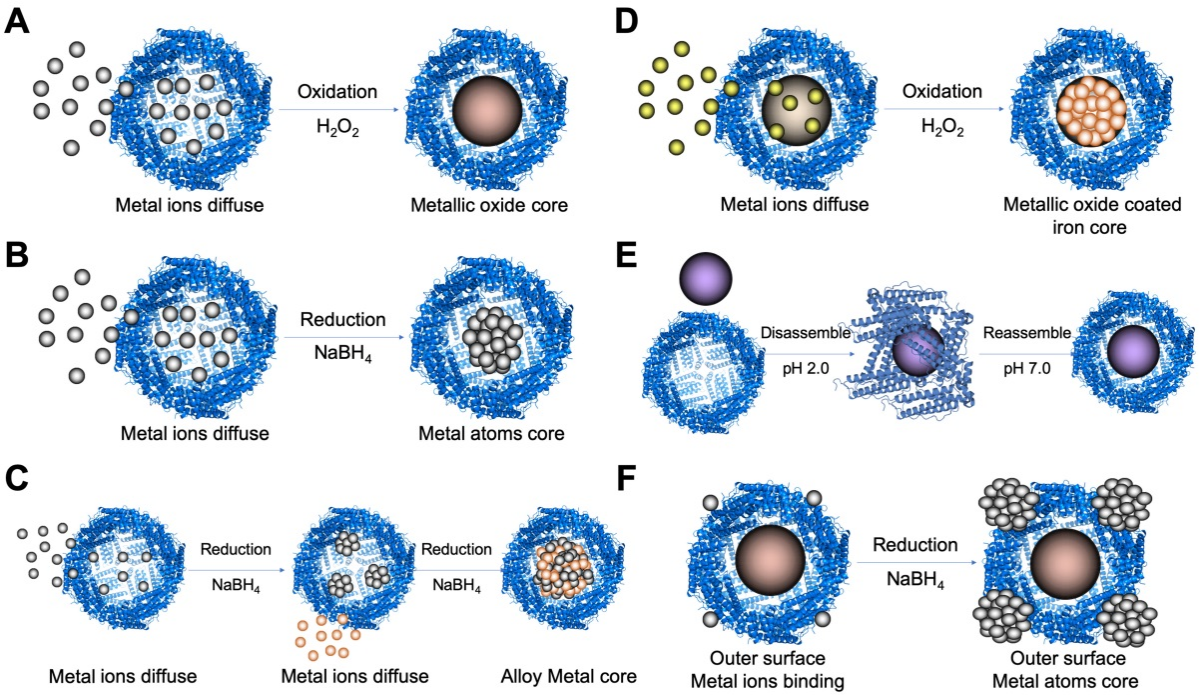
The synthetic strategies of ferritin nanozymes. (A) Oxidation-based biomineralization of ferritin nanozymes. (B) Reduction-based synthesis of ferritin nanozymes. (C) Two-step reduction-based synthesis of ferritin nanozymes. (D) Oxidation and deposition-based synthesis of ferritin nanozymes. (E) pH-dependent disassembly-reassembly based synthesis of ferritin nanozymes. (F) Outer surface reduction-based synthesis of ferritin nanozymes. (PDB entry 5N27).

Due to the superiority of ferritin nanocage as a nanoreactor to synthesize nanomaterials with controllable size, some studies employed ferritin to synthesize nanozymes in its protein cavity by different methods (Table 2) (Figure 6).

Metal ions oxidation (Figure 6 A) or reduction (Figure 6 B) in the cavity of ferritin was the most common method to synthesize ferritin nanozymes. Through two-step reduction reactions, some alloy metal-based ferritin nanozymes were also synthesized (Figure 6 C). Moreover, by directly oxidizing metal ions on the surface of ferrihydrite core in the cavity of ferritin, ferritin nanozymes were synthesized (Figure 6 D). In addition, through a pH-dependent disassembly-reassembly procedure of ferritin, some nanozymes were incorporated into the cavity of ferritin (Figure 6 E). In addition, some metal ions bound on the outer surface of ferritin protein cages. After the addition of reducing agents, metal nanozymes were synthesized on the outer surface of ferritin (Figure 6 F). Taken together, these ferritin nanozymes synthesized in different methods showed different kinds of enzyme-like activities and exerted different functions in applications.

#### 3.2.1 Ferritin nanozymes with ferroxidase-like activity

Since the ferritin L-subunit lacks ferroxidase site, the recombinant L-subunit ferritin (LFn) doesn't possess the iron storage ability. In 2014, L. Li *et al* reported that recombinant human LFn can obtain ferroxidase-like activity by synthesizing Pt nanoparticles in its cavity through a reduction process 65 (Figure 6 B). The synthesized LFn-Pt ferritin nanozymes can catalyze the oxidation of Fe^2+^ ions and acquire the iron storage ability, thus regulating the iron homeostasis in a cellular environment 65.

In addition, ferritin nanozymes can be used for enhancing ferroxidase-like activity of ferritin. Through a reduction process, Au, Ag, Pt nanoparticles were synthesized in the cavity of HSAF. The obtained HSAF-Au 66, HSAF-Ag 66, HSAF-Pt 67 ferritin nanozymes showed enhanced ferroxidase-like activity than that of HSAF. A series of experiments have showed that these nanozymes improved the iron uptake ability of ferritin *in vivo* 66, 67.

#### 3.2.2 Ferritin nanozymes with peroxidase-like activity

Some types of ferritin nanozymes were reported to show peroxidase-like activity. After the discovery of the peroxidase-like activity of Fe_3_O_4_ nanoparticles 1, our group realized that the biomimetically mineralized Fe_3_O_4_ core in the cavity of ferritin nanocages may also show peroxidase-like activity. Later, we synthesized Fe_3_O_4_ core (4.7 ± 0.8 nm) in the cavity of recombinant human H-subunit ferritin (HFn) 12. This HFn-Fe_3_O_4_ nanozyme was tested for tumor diagnosis by exerting its peroxidase-like activity 12.

Since then, many metal oxide nanoparticles, such as Fe_3_O_4_ 17, 68, 69, Co_X_Fe_3-X_O_4_ 69, Co_3_O_4_ 16 were reported to be biomimetically mineralized and deposited in the cavity of ferritin nanocages and showed peroxidase-like activity. By loading Fe^2+^ and Co^2+^ ions simultaneously, a series of Co_X_Fe_3-X_O_4_ mineral cores were formed in the cavity of HFn 69. The size of these Co_X_Fe_3-X_O_4_ mineral cores were controlled around 6 nm with narrow distribution 69. And these synthesized HFn-Co_X_Fe_3-X_O_4_ ferritin nanozymes showed higher peroxidase-like activity than that of HFn-Fe_3_O_4_ nanozymes 69.

Furthermore, one of our works showed that Co_3_O_4_ cores were formed in the cavity of *pyrococcus furiosus* ferritin (pfFn) by mimicking the iron loading process of ferritin 16 (Figure 6 A). We compared the catalytic activity of pfFn-Co_3_O_4_ and pfFn-Fe_3_O_4_ ferritin nanozymes by calculating the *K*_cat_/*K*_M_, which reflects the catalytic efficiency of an enzyme for a given substrate. The as-prepared pfFn-Co_3_O_4_ ferritin nanozymes showed much higher peroxidase-like activity than that of pfFn-Fe_3_O_4_ ferritin nanozymes, as the *K*_cat_/*K*_m_ of pfFn-Co_3_O_4_ ferritin nanozymes was 20-fold higher than that of pfFn-Fe_3_O_4_ ferritin nanozymes for substrates TMB and H_2_O_2_, respectively 16.

Lucia Melnikova *et al* reported that Fe_3_O_4_/γ-Fe_2_O_3_ nanozymes were biomimetically synthesized in the cavity of HSAF 68. HSAF- Fe_3_O_4_/γ-Fe_2_O_3_ possesses peroxidase-like activity which catalyzes the decomposition of H_2_O_2_ in the presence of N,N-diethyl-p-phenylenediamine sulfate (DPD) substrate 68. By increasing the loading amount of Fe^2+^ ions, the size of Fe_3_O_4_/γ-Fe_2_O_3_ magnetic core formed inside HSAF increased from 3.21 nm to 6.83 nm 68. The authors showed that there was a linear dependence between peroxidase-like activity of HSAF- Fe_3_O_4_/γ-Fe_2_O_3_ and the size of Fe_3_O_4_/γ-Fe_2_O_3_ magnetic core 68. Apoferritin exhibits seldom peroxidase-like activity 68, which is consistent with the report of Tang *et al* 41.

It was reported that [Fe(CN)_6_]^4-^ ions reacted with Fe^3+^ ions on the surface of iron oxide core of horse spleen ferritin can form Prussian Blue nanoparticles (PBNPs) on the surface of ferrihydrite within the ferritin cavity under acid environment (pH 3.0) 70 (Figure 6 D). Horse spleen ferritin-PBNPs ferritin nanozymes showed peroxidase-like activity similar with that of Prussian Blue coated Fe_2_O_3_ magnetic nanoparticles, which catalyzes the peroxidase substrates TMB and 2, 2'-azino-bis(3-ethylbenzothiazoline-6-sulfonic acid) (ABTS) to give a color reaction 70. The peroxidase-like activity of horse spleen ferritin-PBNPs nanozymes is pH, temperature dependent and is well fits with the typical Michaelis-Menten kinetics for H_2_O_2_, TMB and ABTS substrates, respectively 70. Horse spleen ferritin-PBNPs ferritin nanozymes exhibited high affinity and sensitivity to H_2_O_2_ when ABTS was used as substrate, which makes horse spleen ferritin-PBNPs ferritin nanozyme a sensitive agent for glucose detection 70.

By loading metal ions into the cavity of ferritin, followed by a reduction process, some metal nanoparticles, such as Au clusters 71, Fe atoms 72 and Fe-Pt, Fe-Pd, Fe-Rh alloy nanoparticles (two-step reduction) 72 (Figure 6 C), were synthesized within the cavity of HSAF and showed peroxidase-like activity. HSAF-Au ferritin nanozyme possessed a 2 nm Au cluster, and exhibited higher peroxidase activity than that of natural enzyme under acidic pH and high temperature (over 60 ℃) 71.

Wei Zhang *et al* reported that when Fe^2+^ ions bound to the inner cavity of HSAF, after adding reductant, 16 Fe atoms were reduced in the cavity of HSAF 72. This HSAF-Fe ferritin nanozyme exhibited peroxidase-like activity and was used for the detection of H_2_O_2_ 72. By continuing loading other metal ions, such as Pt, Rh and Pd, mineral cores (Fe-Pt, Fe-Rh, Fe-Pd) were formed in the cavity of HSAF with narrow size distribution (around 6 nm) 72 (Figure 6 C). The synthesized HSAF-Fe-Pt, HSAF-Fe-Rh and HSAF-Fe-Pd ferritin nanozymes showed much higher peroxidase-like activity than that of HSAF-Fe ferritin nanozymes 72.

Some kinds of noble metal ions also anchored the outer surface of ferritin nanocages, thus formed non-coated metal nanoparticles possess enzymatic activity. For example, Marie Peskova *et al* reported that Ag and Pd ions bound to the outer surface of *Pyrococcus furiosus* ferritin (pfFn) 73. By controlling the amount of metal ions and reductants in the reaction system, non-coated Ag and Pd nanoparticles were synthesized on the outer surface of pfFn with a confined size distribution (4.7 ± 1.59 nm for Ag, 4.21 ± 1.39 nm for Pd) 73 (Figure 6 F). The synthesized pfFn-Ag and pfFn-Pd ferritin nanozymes possess peroxidase-like activity and their enzymatic activity was impacted by detergents 73. The peroxidase-like activity of pfFn-Ag ferritin nanozymes was only detected in the presence of sodium dodecyl sulfate (SDS), while peroxidase-like activity of pfFn-Pd ferritin nanozymes was enhanced by SDS and other anionic detergents 73.

#### 3.2.3 Ferritin nanozymes with oxidase-like activity

S. Kanbak-Aksu *et al* reported that Pt nanoclusters with a size distribution of 5 ± 1 nm were synthesized within the cavity of pfFn through a reduction process 74 (Figure 6 B). The synthesized pfFn-Pt ferritin nanozymes showed oxidase-like activity which catalase the aerobic oxidation of alcohols in the presence of O_2_ 74. The excellent thermal stability and high hydrophilicity of pfFn makes pfFn-Pt ferritin nanozyme a reliable catalyst for aerobic oxidation of alcohols at high temperatures or other harsh environments.

#### 3.2.4 Ferritin nanozymes with superoxide dismutase- (SOD-) like activity

Taken advantages of the pH dependent disassembly-reassembly feature of ferritin, some nanozymes, such as nano-CeO_2_ 75, can be encapsulated in the cavity of ferritin nanocages. Under acid condition, pH 2.0, ferritin nanocages disassembled into subunits, once the pH return to physiological condition, ferritin subunits reassembled into a protein nanocage. Thus, CeO_2_ nanoparticles can be encapsulated into the cavity of HSAF nanocages during the reassembly process 75 (Figure 6 E). HSAF-CeO_2_ ferritin nanozymes exhibited higher SOD-like activity which scavenging reactive oxygen species (ROS) than that of CeO_2_ nanozymes compared under the same concentration of Ce atom.

#### 3.2.5 Ferritin nanozymes with multi-enzyme-like activities

Some kinds of ferritin nanozymes showed multi-enzyme-like activities. For example, Jia Fan *et al* reported that a 1.87 ± 0.40 nm Pt nanoparticle was synthesized within the cavity of HSAF by reduction 76 (Figure 6 B). HSAF-Pt ferritin nanozymes exhibit peroxidase and catalase activities in a pH and temperature dependent manner 76. In acidic pH, HSAF-Pt ferritin nanozymes mainly showed peroxidase-like activity, and the optimum pH was 4.0, same as that of natural HRP. In neutral and alkaline pH, HSAF-Pt ferritin nanozyme mainly showed catalase-like activity, and the catalase-like activity enhanced with the increase of pH (from pH 7.0 to pH 12.0) 76. Both the peroxidase- and catalase-like activities of HSAF-Pt ferritin nanozymes were maintained even when the temperature was over 60 ℃ 76. Moreover, when the temperature increased from 20 ℃ to 80 ℃, the catalase-like activity of HSAF-Pt ferritin nanozymes increased gradually, while the peroxidase-like activity of HSAF-Pt ferritin nanozymes decreased gradually 76. In addition, the authors showed that HSAF alone, which is a protein shell without iron, did not possess peroxidase-like activity 76, indicating that the peroxidase-like activity comes from the inner Pt nanoparticles, not the ferritin protein shell. This conclusion is consistent with the report by Tang et al in 2011 41.

Moreover, Lianbing Zhang *et al* showed that HSAF-Pt ferritin nanozymes exhibited both catalase- and SOD-like activities under pH 7.4 physiological environment 77. The catalase- and SOD-like activities of HSAF-Pt ferritin nanozymes cooperated together to eliminate intercellular ROS. Later on, they synthesized a 3 nm PtAu alloy nanoparticle inside the HSAF by reduction, and they showed that HSAF-PtAu ferritin nanozymes possess higher catalase- and SOD-like activities than that of HSAF-Pt ferritin nanozymes 78.

Au-Ag alloy nanoparticles were also reported to be synthesized in the cavity of HSAF by reduction 79, 80. Changing the ratio of Au atoms / Ag atoms in the reaction system did not affect the uniform size of Au-Ag alloy nanoparticles (around 6 nm) inside the HSAF 80. The synthesized HSAF-AuAg ferritin nanozymes showed catalytic activity on the reduction of 4-nitrophenol 80. Soon afterwards, it was reported that HSAF-AuAg ferritin nanozymes exhibited peroxidase-, catalase-, and SOD-like activities simultaneously, which was used for cytoprotecting 79. The authors showed that these three kinds of enzyme-like activities mainly generated from Au-Ag alloy nanoparticles, while the HSAF protein shell accelerates the electron transfer and promote the catalytic process 79.

Kelong Fan* et al* reported that Nitrogen-doped Porous Carbon Nanospheres (N-PCNSs) possess four enzyme-like activities (oxidase, peroxidase, catalase and SOD) 81. By chemical coupling recombinant Human HFn nanocages on the outer surface of N-PCNSs nanozymes (100 ± 10 nm), a ferritin nanozyme (HFn-N-PCNSs) was synthesized 81.

#### 3.2.6 Ferritin nanozymes with other enzyme-like activities

Some artificial metalloenzymes were also reported to be encapsulated or in site synthesized in the cavity of ferritin nanocages. For example, Artificial metalloenzyme [Cp*Ir(biot-p-L)Cl]·Sav (with a size distribution of 4.5 nm · 5.5 nm · 5.1 nm) was encapsulated in the cavity of HSAF by pH dependent disassembly-reassembly method 82. This ferritin nanozyme possesses artificial hydrogenase activity, which catalyzes the reduction of cyclic imines into nontoxic product 82.

Another work showed that artificial metalloenzyme [Rh(nbd)Cl]_2_ can be in site immobilized in the inner cavity of recombinant horse LFn 83. This ferritin nanozyme possesses catalytic activity for polymerization of phenylacetylene 83.

These works suggested that ferritin nanozyme may be an ideal strategy for protecting artificial metalloenzymes from the harsh environment and *in vivo* delivery of artificial metalloenzymes.

## 4. Applications of ferritin nanozymes

### 4.1 Biosensing

#### 4.1.1 H_2_O_2_ detection

HSAF-Fe_3_O_4_/γ-Fe_2_O_3_ ferritin nanozyme was used for H_2_O_2_ detection by exerting its peroxidase-like activity 68. HSAF-Fe_3_O_4_/γ-Fe_2_O_3_ catalyzes the oxidation of substrate DPD to the radical cation DPD^+^ in the presence of H_2_O_2_. DPD^+^ produces a stable purple color with peak absorption at 551 nm, the amount of H_2_O_2_ added in the reaction system determined this absorption. By plotting the standard curve between the increase of absorption value per minute (dA/min) and H_2_O_2_ concentration, the concentration of H_2_O_2_ can be determined in the range of 5.8-88.2 mM 68. Since the oxide TMB produced a blue color with peak absorption at 652 nm, HSAF-Fe ferritin nanozymes were reported to use TMB as substrate to detect H_2_O_2_ 72.

#### 4.1.2 Glucose detection

The catalytic oxidation of glucose by oxygen in the presence of glucose oxidase (GOx) produces H_2_O_2_. Determining the concentration of H_2_O_2_ produced in this reaction indirectly reflects the concentration of glucose. Ferritin nanozymes possessing peroxidase-like activity, such as HSAF-PBNPs 70, HSAF-Au 71, HSAF-Fe 72, were used for glucose detection in this way. Due to the high affinity of HSAF-PBNPs to H_2_O_2_, the glucose can be detected as low as 0.39 µM with the linear range of 0.39 µM to 6.25 µM 70. HSAF-PBNPs based glucose detect system was more sensitive than that of naked GO-COOH (1 µM) and Fe_3_O_4_ (5 µM) nanozymes 70, indicating the superiority of ferritin nanozymes over naked nanozymes in biosensing.

#### 4.1.3 Enzyme-linked immunosorbent assay (ELISA)

HSAF-PBNPs ferritin nanozymes were also used for ELISA to detect anti-horse spleen ferritin antibodies 70. The positive results also indicated that the loading of nanozymes does not affect the targeting ability of ferritin protein shell.

### 4.2 Disease diagnosis

#### 4.2.1 Tumor diagnosis

**Figure 7:**
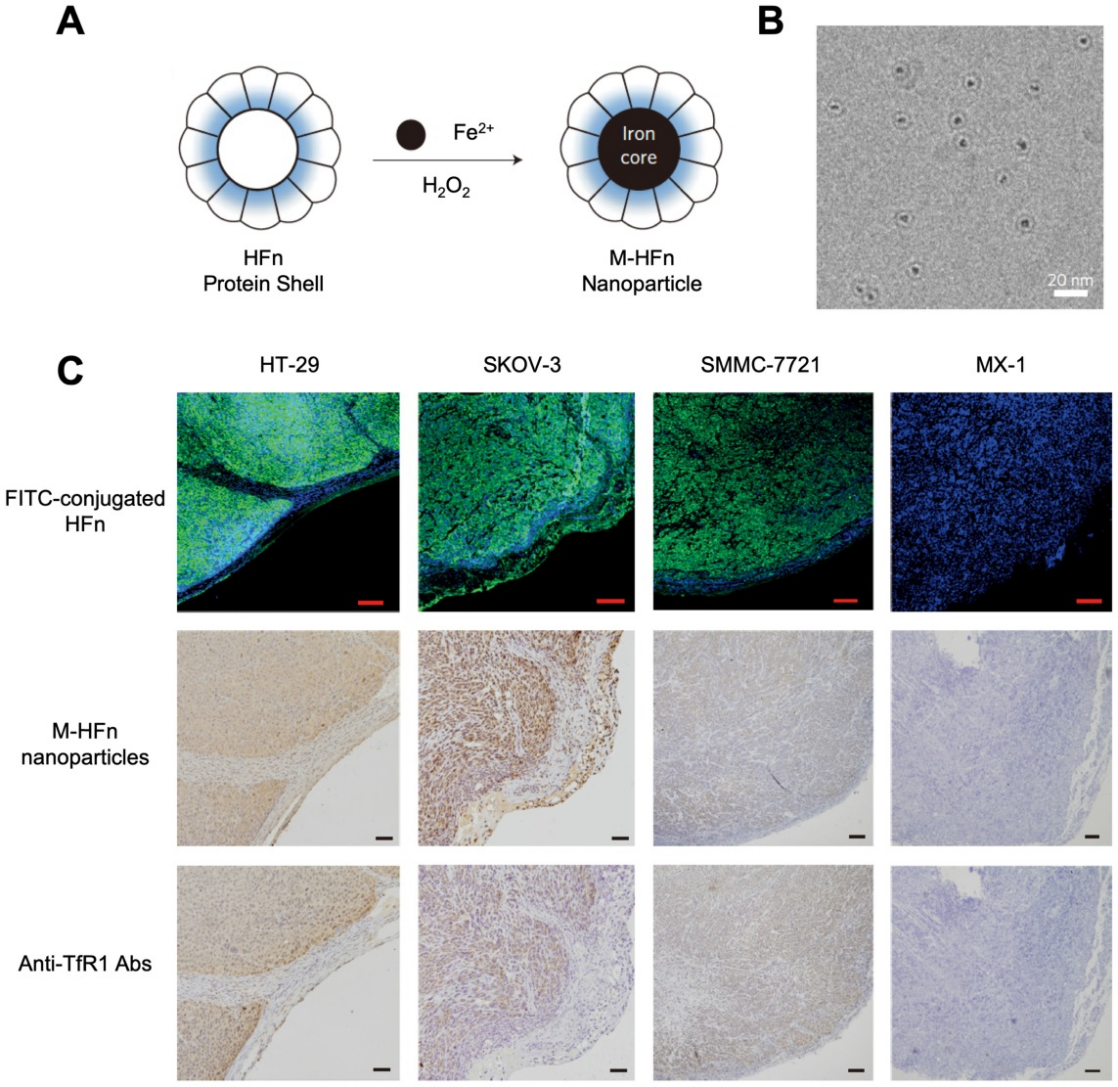
(A) The synthesis process of HFn-Fe_3_O_4_ ferritin nanozymes. (B) Cryo TEM image of HFn-Fe_3_O_4_ ferritin nanozymes. Scale bar = 20 nm (C) HFn-Fe_3_O_4_ ferritin nanozymes target and visualize xenografted human tumor tissues. Human colorectal cancer cell line HT-29, human ovarian carcinoma cell line SKOV-3, hepatocellular carcinoma cell line SMMC-7721 are TfR1-positive cells, human breast cancer cell line MX-1 are TfR1-negative cells. In FITC-conjugated HFn group, green signal represented FITC, blue signal represented DAPI-stained nuclei. In M-HFn and Anti-TfR1 Abs groups, the tissues were stained by DAB (yellowish-brown signal), and then counterstained by hematoxylin (light blue signal). Reprinted with permission from 12. Copyright 2012 Springer Nature.

In 2012, for the first time, Kelong Fan *et al* reported that HFn-Fe_3_O_4_ ferritin nanozyme was a dual-function agent for targeting and visualizing tumor tissues 12 (Figure 7). Fe_3_O_4_ nanoparticles were biomimetically synthesized in the cavity of recombinant human HFn. HFn specifically binds to tumor cells which overexpress transferrin receptor 1 (TfR1) 9, 12-15, while the Fe_3_O_4_ core exerts peroxidase-like activity to catalyze the oxidation of substrate di-azo-aminobenzene (DAB) in the presence of H_2_O_2_ to give a color reaction. Ferritin endows nanozyme tumor targeting ability. This dual-function of HFn-Fe_3_O_4_ ferritin nanozyme makes it a one-step agent for rapid pathological diagnosis of tumor tissues. HFn-Fe_3_O_4_ ferritin nanozyme possesses advantages (such as time-saving, low cost, ease to operate et al) over traditional HRP-conjugated antibodies for tumor diagnosis. The authors examined 474 clinical specimens from patients with 9 types of tumors, and they verified that ferritin nanozymes can distinguish tumor cells from normal cells with a sensitivity of 98% and specificity of 95%.

In 2017, Tongwei Zhang *et al* reported that by substituting HFn-Fe_3_O_4_ into cobalt-doped HFn-Co_X_Fe_3-X_O_4_ ferritin nanozyme, its peroxidase-like activity was enhanced 69. The authors synthesized a series of Co_X_Fe_3-X_O_4_ cores with different cobalt-doping ratios (0, 20%, 40%, 60%) inside the HFn protein shell. They found that the peroxidase-like activity of HFn-Co_X_Fe_3-X_O_4_ ferritin nanozymes was positively correlated with the cobalt-doping ratio of Co_X_Fe_3-X_O_4_ core. The peroxidase-like activity of ferritin nanozymes relied on its mineral core. Enhanced peroxidase-like activity of HFn-Co_X_Fe_3-X_O_4_ ferritin nanozymes made it a more sensitive agent for tumor diagnosis.

**Figure 8:**
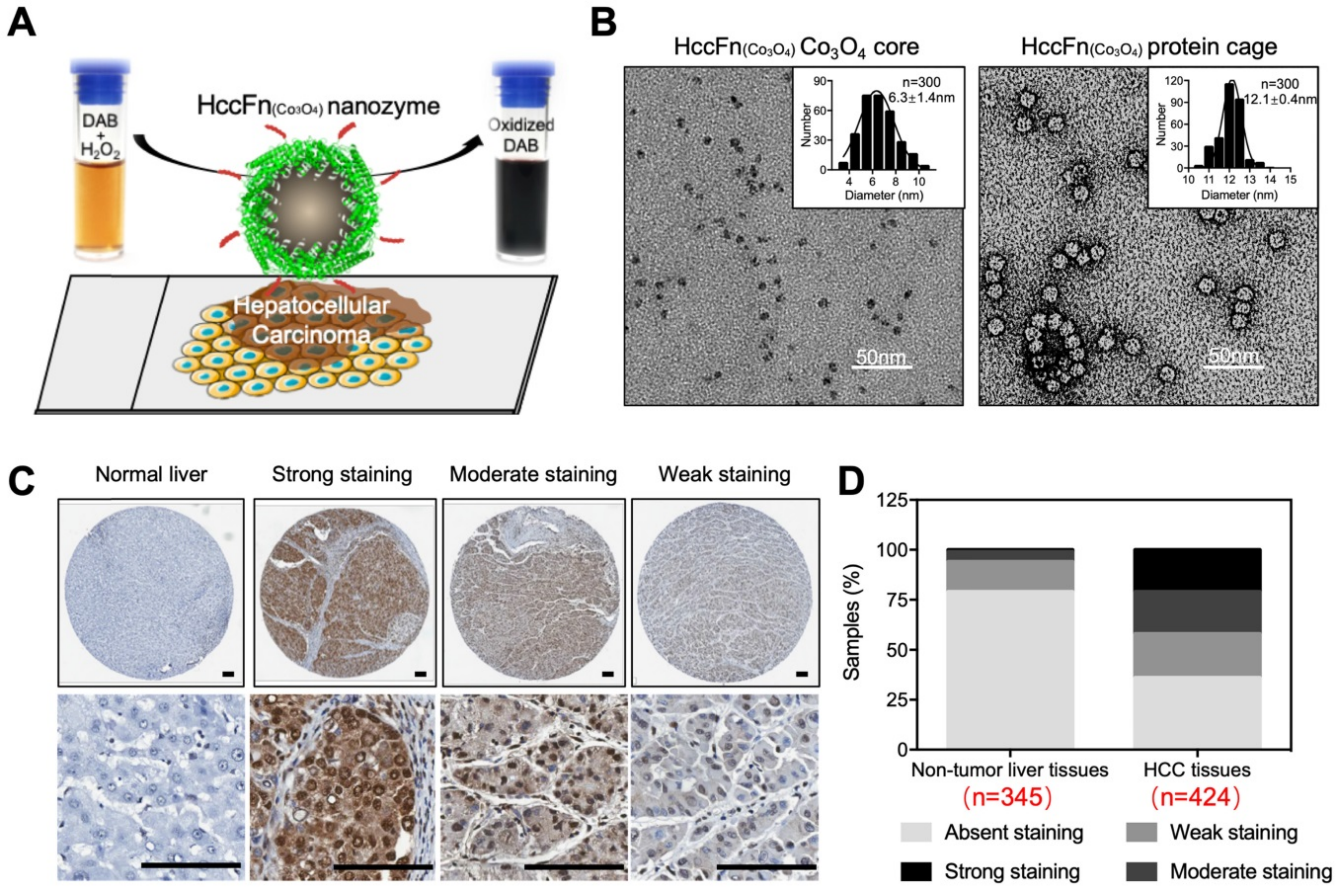
HccFn-Co_3_O_4_ ferritin nanozyme-based HCC diagnosis. (A) Schematic diagram of HccFn-Co_3_O_4_ ferritin nanozyme-based HCC diagnosis. (B) Transmission electron microscope (TEM) images of Co_3_O_4_ core and protein cage of HccFn-Co_3_O_4_ ferritin nanozyme. The size distribution of respond nanoparticles were shown on the top right corner. Scale bar = 50 nm. (C) HccFn-Co_3_O_4_ ferritin nanozyme based staining in tissue microarrays derived from HCC patients. The normal liver tissues and HCC tissues were stained by DAB (yellowish-brown signal), and then counterstained by hematoxylin (light blue signal). (D) Staining analysis of HccFn-Co_3_O_4_ ferritin nanozyme to non-tumor liver tissues and HCC tissues. Reprinted with permission from 16. Copyright 2019 American Chemical Society.

In 2019, Bing Jiang *et al* reported that Co_3_O_4_ core was biomimetically synthesized in the cavity of hepatocellular carcinoma (HCC) targeted peptide (SP94) displayed pfFn (HccFn) nanocages 16. The synthesized ferritin nanozyme was named HccFn-Co_3_O_4_. HccFn ferritin nanocages possess HCC-targeted ability, which is enabled by the display of HCC cell-specific peptide SP94 on the outer surface of pfFn through a genetic engineering approach. The peroxidase-like activity of Co_3_O_4_ core catalyzes the substrate to make color reaction to visualize HCC tumor tissues. Due to the apparent higher peroxidase-like activity of Co_3_O_4_ core than Fe_3_O_4_ core (20-fold higher catalytic efficiency) and HCC targeted ability of HccFn, HccFn-Co_3_O_4_ ferritin nanozymes showed high sensitivity for HCC-specific diagnosis (Figure 8). The authors examined 424 clinical HCC specimens and verified that HccFn-Co_3_O_4_ ferritin nanozymes could distinguish HCC tissues from normal liver tissues with a sensitivity of 63.5% and specificity of 79.1%, which was comparable with that of clinically used HCC specific marker alpha fetoprotein (AFP). Moreover, the pathological analysis indicated that HccFn-Co_3_O_4_ ferritin nanozymes staining result was a potential predictor of prognosis in HCC patients. Staining intensity was positively correlated to tumor differentiation degree (P = 0.0246), tumor invasion (P < 0.0001) and negatively correlated with overall survival (P = 0.0084) of HCC patients.

#### 4.2.2 Cardiovascular disease diagnosis

**Figure 9:**
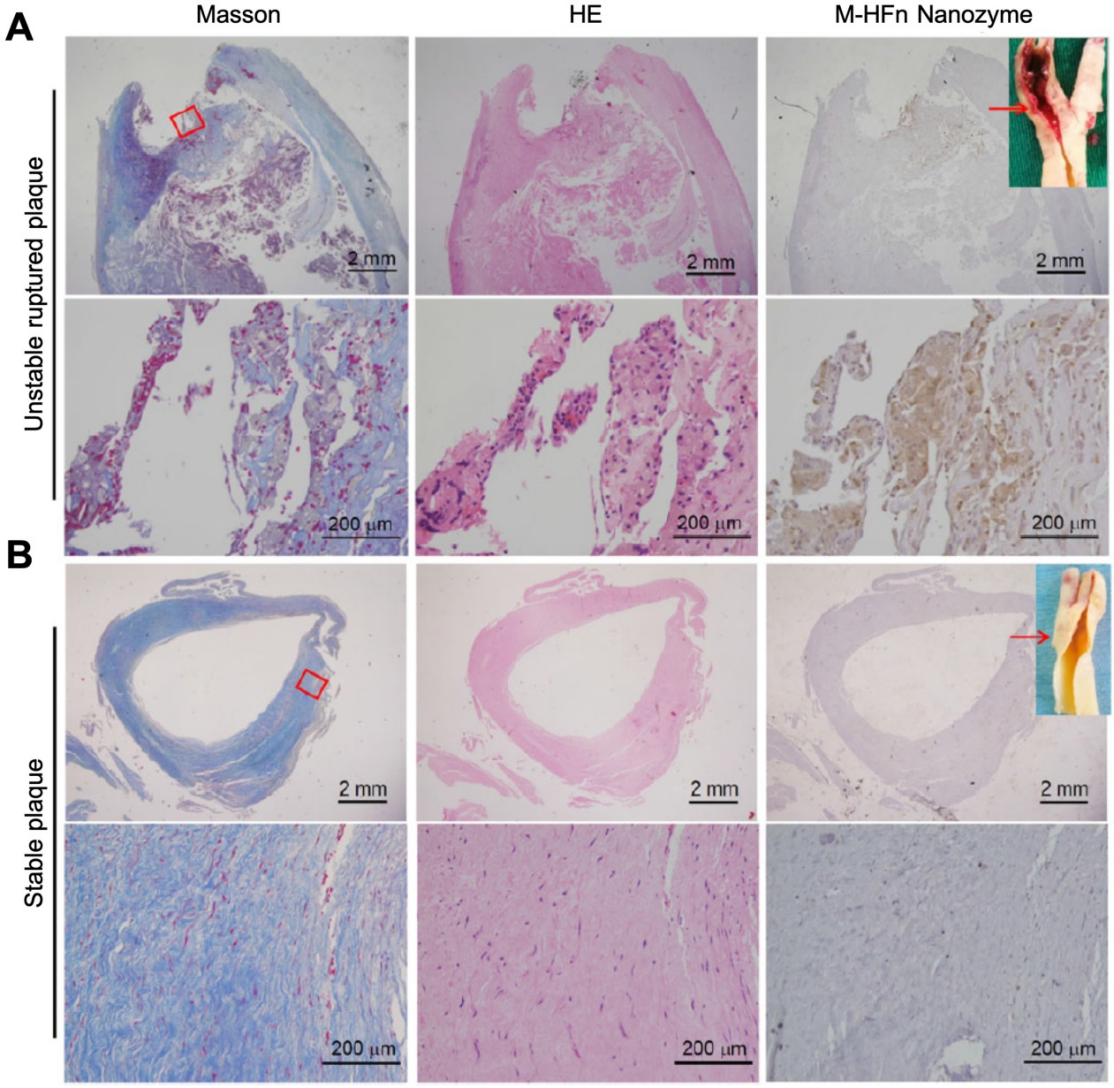
HFn-Fe_3_O_4_ ferritin nanozymes specifically stain unstable and ruptured plaques 17. Carotid sections with unstable ruptured plaque (A) and stable plaque (B) were attained by Masson (for collagen distribution identification), HE and HFn-Fe_3_O_4_ ferritin nanozymes (stained by DAB, and then counterstained by hematoxylin). Arrows indicate the histological section position. Reprinted with permission from 17. Copyright 2019 Tsinghua University Press and Springer-Verlag GmbH Germany, part of Springer Nature.

HFn-Fe_3_O_4_ ferritin nanozymes were further explored to be used for pathological identification of high-risk and ruptured atherosclerotic plaques in humans 17. The authors showed that HFn could intrinsically recognize plaque-infiltrated active macrophages through the interaction with TfR1. Plaque-infiltrated active macrophages drive atherosclerotic plaque progression and rupture and are significantly associated with the plaque vulnerability, thus HFn-Fe_3_O_4_ ferritin nanozymes specifically stain unstable ruptured plaque (Figure 9). Stable plaque seldom exists plaque-infiltrated active macrophages, thus HFn-Fe_3_O_4_ ferritin nanozymes did not stain stable plaque.

### 4.3 Cancer Therapy

Ferritin has been widely used as a nanocarrier to deliver various drugs for cancer therapy. Due to its unique self-assemble ability, hollow cavity capable of encapsulating drugs, and an outer surface that can be modified genetically and chemically for targeting, ferritin has recently emerged as a promising drug delivery carrier 84. Many chemotherapeutic drugs and small molecules, such as doxorubicin 85, cisplatin 86, curcumin 87, gefitinib 88, siRNA 89, have been encapsulated into ferritin for cancer therapy. However, by exerting the enzyme-like activities of ferritin nanozymes to kill tumor cells was rarely being reported. As described below, we introduce a ferritin nanozyme which combines the tumor targeting ability of ferritin with the multi-enzyme-like activities of nanozyme to treat tumor.

**Figure 10:**
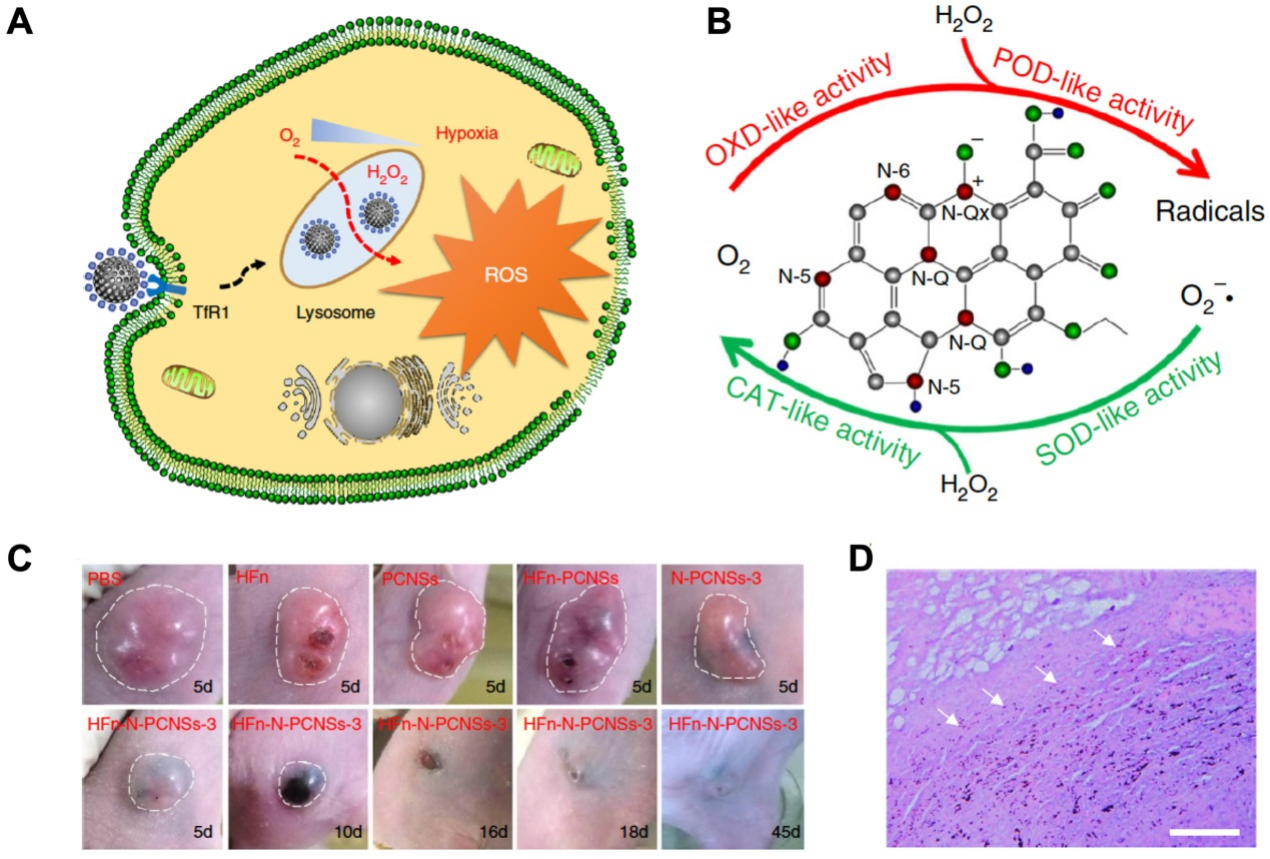
HFn-N-PCNSs ferritin nanozymes-based cancer therapy. (A) Schematic diagram for HFn-N-PCNSs ferritin nanozymes induced tumor cell death. (B) HFn-N-PCNSs ferritin nanozymes show four enzyme-like activities. (C) Tumor response to HFn-N-PCNSs ferritin nanozymes treatment. (D) Accumulation of HFn-N-PCNSs ferritin nanozymes in tumor tissues. White arrows indicate the location of HFn-N-PCNSs ferritin nanozymes. Reprinted with permission from 81. Copyright 2018 Springer Nature.

In 2018, Kelong Fan *et al* reported that HFn-conjugated Nitrogen-doped Porous Carbon Nanospheres (HFn-N-PCNSs ferritin nanozymes) possess four enzyme-like activities (oxidase, peroxidase, catalase and SOD), which are responsible for ROS regulation 81. Due to the tumor targeting and lysosome localization ability of HFn and intracellular ROS generation ability of N-PCNSs nanozymes under acidic pH, HFn-N-PCNS ferritin nanozymes were evaluated for tumor targeted catalytic therapy 81. HFn specifically bound to the TfR1 overexpressed on the surface of tumor cells, then HFn was endocytosed into lysosomes where the pH environment is acidic. Once the HFn-N-PCNSs ferritin nanozymes localized to the acidic pH environment of tumor lysosomes, it mainly displayed peroxidase- and oxidase-like activities. These two enzyme-like activities allow it to exert ROS mobilization including (1) transferring oxygen to free radicals accompanied with O_2_ consumption, and (2) catalyzing H_2_O_2_ into free radicals, which thus synergistically burst ROS level and induce cell damage. The excess ROS generated in tumor cells caused tumor regression (Figure 10).

### 4.4 Antioxidation and cell protection

**Figure 11:**
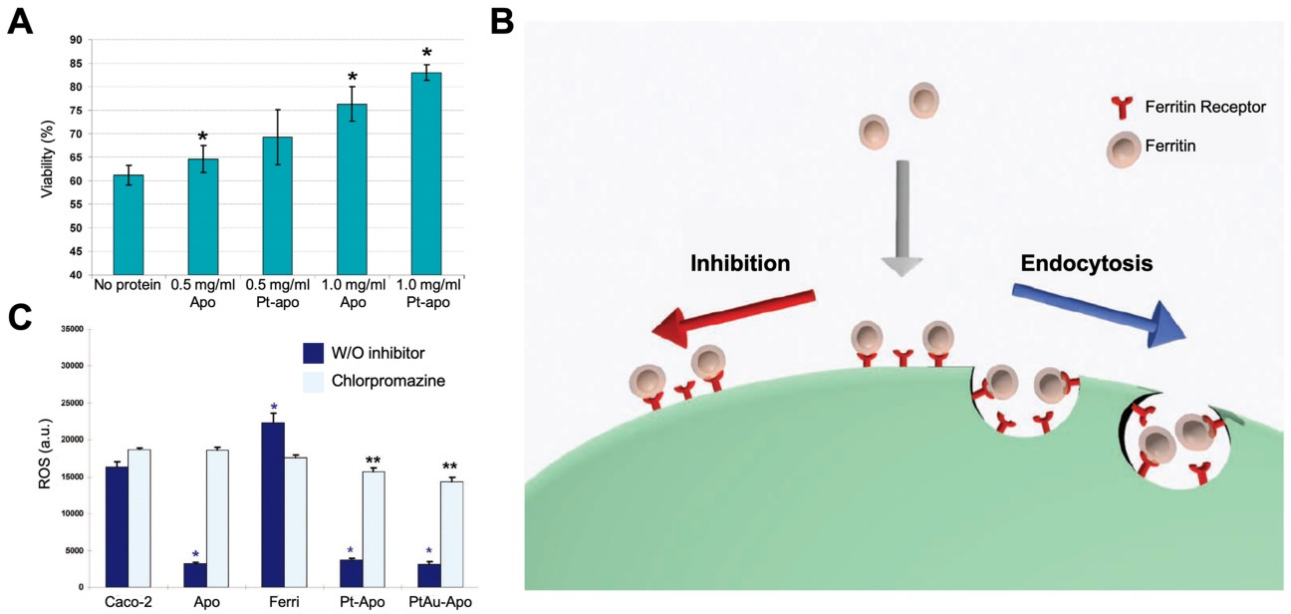
(A) Effects of treatment with HSAF-Pt ferritin nanozymes on the viability of Caco-2 cells stressed with 5 mM H_2_O_2_. Reprinted with permission from 77. Copyright 2010 American Chemical Society. (B) The binding and cellular uptake of ferritin nanozymes via receptor-mediated endocytosis. This endocytosis was blocked by inhibitors. Reprinted with permission from 78. Copyright 2011 WILEY‐VCH Verlag GmbH & Co. KGaA, Weinheim. (C) Effects of treatment with HSAF-Pt and HSAF-PtAu ferritin nanozymes on the ROS generation of Caco-2 cells stressed with 2 mM H_2_O_2_. Chlorpromazine was used to inhibit endocytosis of ferritin. Reprinted with permission from 78. Copyright 2011 WILEY‐VCH Verlag GmbH & Co. KGaA, Weinheim.

Ferritin nanozymes were used for antioxidation and cell protection *in vivo* by exerting its enzyme-like activities to eliminate ROS. In 2010, Lianbing Zhang *et al* reported that HSAF-Pt ferritin nanozymes exhibited catalase- and SOD-like activities under pH 7.4 physiological environment 77. When incubating with Caco-2 cells stressed with 5 mM H_2_O_2_, HSAF-Pt ferritin nanozymes protected cells from ROS damages and significantly increase the cell viability (Figure 11 A). Interestingly, the HSAF also showed cell protection ability, which indicated that apoferritin may also possess the ability to eliminate ROS. Later on, they found that HSAF-PtAu ferritin nanozymes exhibited higher catalase- and SOD-like activities than that of HSAF-Pt, and showed better effect on cell protection 78. The authors also supposed that HSAF possesses a receptor on cell surface. Through receptor-mediated endocytosis, ferritin nanozymes may enter into cells and exert enzyme-like activities to eliminate intracellular ROS (Figure 11 B). This hypothesis was later verified, as the authors found that HSAF-Pt and HSAF-PtAu ferritin nanozymes significantly reduced the intracellular ROS of Caco-2 cells stressed with 2 mM H_2_O_2_. After the treatment of endocytosis inhibitor chlorpromazine, the intracellular ROS of Caco-2 cells was significantly elevated (Figure 11 C).

Moreover, HSAF-Au-Ag ferritin nanozymes were shown to possess triple-enzyme like activities (SOD, catalase, peroxidase) which scavenge O_2_^-^ and reduce H_2_O_2_ to eliminate intracellular ROS 79. HSAF-Au-Ag ferritin nanozymes have pH-dependent peroxidase- or catalase-like activities. In acidic pH environment of lysosome, it would be able to scavenge intracellular H_2_O_2_ by exerting its peroxidase-like activity. However, in other parts of cytoplasm (near neural pH), HSAF-Au-Ag ferritin nanozymes mainly show catalase- and SOD-like activities and scavenge intracellular O_2_^-^ and H_2_O_2_.

Xiangyou Liu et al also reported that HSAF-CeO_2_ ferritin nanozymes possess SOD-like activity which scavenge O_2_^-^ to scavenge ROS 75. The authors declared that HSAF possesses a receptor on cell surface which mediated the active cellular uptake of HSAF-CeO_2_ ferritin nanozymes. This viewpoint corresponded to that of Lianbing Zhang *et al* reported 78. Due to the improved biocompatibility and active endocytosis process initiated by HSAF protein cages, HSAF-CeO_2_ ferritin nanozymes significantly scavenge the intracellular ROS of hepatoma carcinoma cell HepG2.

Although the above works did not clarify what the ferritin receptors are, many works have reported the receptors of different types of ferritins. The receptor of HSAF was reported to be mouse Scara5 by many groups, same as that of mouse LFn 90-93. The receptor of human HFn was reported to be human TfR1 9, 12-15, while the receptor of the mouse HFn was mouse TIM-2 94-96. In addition, human HFn also recognized mouse TIM-2 due to a cross reaction 90, 97. Moreover, it was recently reported that human oligodendrocytes took up human HFn to meet iron requirement via the human Tim-1 receptor 98. The receptor of human LFn in the human body is still unknown. If the ferritin was only recognized by the corresponding receptor and entered into cells through receptor-mediated endocytosis, so that ferritin nanozymes exert its ROS regulation function.

### 4.5 Other applications

**Figure 12:**
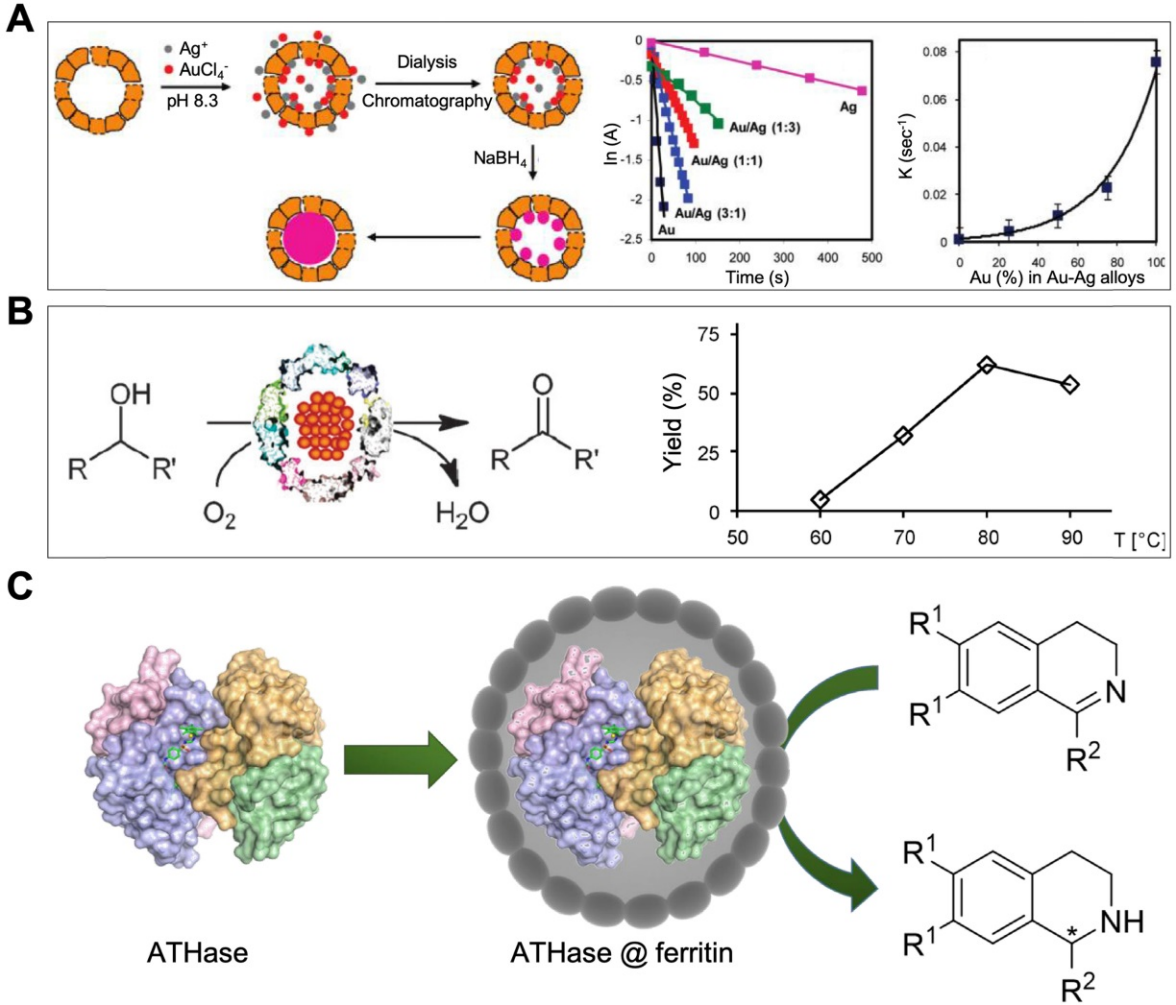
(A) HSAF-Au-Ag ferritin nanozymes catalyze the reduction of 4-nitropheno. The catalytic reaction rate is positively correlated with the Au/Ag ratio of Au-Ag alloy nanoparticles. Reprinted with permission from 80. Copyright 2010 American Chemical Society. (B) pfFn-Pd ferritin nanozymes catalyze the aerobic oxidation of alcohols. Reprinted with permission from 74. Copyright 2012 Royal Society of Chemistry. (C) ATHase@ferritin ferritin nanozymes catalyze the reduction of cyclic imines. Reprinted with permission from 82. Copyright 2018 Royal Society of Chemistry.

Ferritin nanozymes were also used for decontaminations. The catalytic activities of ferritin nanozymes can oxidase or reduce organic reagents which are harmful to human bodies or environment. By encapsulating Au-Ag alloy nanoparticles inside the HSAF, HSAF-Au-Ag ferritin nanozymes were synthesized 80. HSAF-Au-Ag ferritin nanozymes catalyzed the reduction of 4-nitrophenol efficiently. This catalytic reaction rate was positively correlated with the Au/Ag ratio of Au-Ag alloy nanoparticles (Figure 12 A), indicating the catalytic activity mainly derives from Au nanoparticles.

In addition, pfFn-Pd ferritin nanozymes were reported to possess oxidase-like activity which catalyze the aerobic oxidation of alcohols 74 (Figure 12 B). This optimum reaction temperature was around 80 ℃. At this temperature, most of natural enzymes were soon denatured, while pfFn-Pd ferritin nanozymes still kept high stability and catalytic activity due to the excellent thermal stability of pfFn. pfFn-Pd ferritin nanozymes also catalyzed the discoloration of diazo dyes and reduction of p-nitrophenol 73.

Moreover, pfFn nanozymes were also used for removing phosphate and arsenate from aqueous solution 99. The pfFn mineral iron cores have strong absorption ability for phosphate and arsenate. Specially, during or after the iron oxidation process catalyzed by pfFn nanozymes and with incorporating phosphate in the reaction system, a ferric-oxyhydroxide-phosphate complex was synthesized inside the cavity of pfFn. pfFn nanozymes removed these oxo anions from aqueous solution to residual pM levels, demonstrating a great potential to be used for sewage treatment.

Moreover, an artificial transfer hydrogenase [Cp*Ir(biot-p-L)Cl]·Sav was also encapsulated into the cavity of HSAF 82. This synthesized ATHase@ferritin ferritin nanozyme catalyzed the reduction of cyclic imines for detoxification (Figure 12 C). HSAF provides protective protein shell for precious metal-based artificial enzymes to defend the complicated physiological environment and possibly deliver it to targeted site *in vivo*.

## 5. Conclusion and perspectives

Since the first discovery of the peroxidase-like activity of Fe_3_O_4_ nanoparticles, nanozyme has been investigated for over a decade. Hundreds of new nanozymes have been developed and applied for biosensing, environmental protection, anti-biofouling, imaging and theranostics. However, the size-controllable synthesis and targeting modifications of nanozymes are still challenging in this filed.

The emerging of ferritin nanozymes provides an effective way to address these challenges. Ferritins are natural nanozymes which exhibit multi enzyme-like activities (*e.g.* ferroxidase, peroxidase). In addition, ferritins are artificial nanozymes which serve as nanoreactors to synthesize nanozymes in its cavity. Compared with other types of nanozymes, ferritin nanozymes possess following prominent advantages:

1) Size-controllable synthesis of nanozymes. Ferritin possesses uniform protein shell and natural synthesis ferric oxide (ferrihydrite) nanoparticles within its protein cavity. Learning the way of biomineralization process of ferritin, we may synthesize different types of metal or metallic oxide nanozymes in the cavity of ferritin with a confined size (less than 8 nm). In addition, the size of nanozymes will be controlled by controlling the amount of metal ions added to the reaction system.

2) Targeting modification of nanozymes. Through genetic or chemical approaches, we can readily modify ferritin nanocages with targeting moieties. Combining targeting modification of ferritin nanocages with nanozyme encapsulated in ferritin inner cavity, we can easily realize targeting modifications of nanozymes. In addition, the size of ferritin nanocage is 12 nm, which is an ideal size for overcoming the physiological barriers posed by the tumor environment and passively target the tumor site. Thus, ferritin nanozymes possess passive tumor targeting ability. Moreover, human HFn can specifically target tumor cells via binding to its receptor TfR1. Some studies reveal that human HFn traverses the blood brain barrier (BBB) 97, 100 , thus it could be used for brain tumor treatment 97, 101. This characteristic endows ferritin nanozymes natural tumor targeting and BBB traversing abilities. Moreover, some nanozymes show pH dependent multi-enzyme-like activities. Under different pH environment *in vivo*, nanozymes may exert distinct functions (generate ROS or eliminate ROS) 102. Through the targeting delivery of ferritin, nanozymes may actively target specific environment and exert special functions.

3) High biocompatibility and stability. Ferritin, as a natural protein, shows excellent biocompatibility for* in vivo* applications. Due to the structure superiority, ferritin exhibits high stability under robust environment, and it can resistant high temperature and denaturants. In addition, benefiting from surrounding ferritin protein as a protective layer, the nanozyme is shielded from nonspecific biomacromolecules cellular binding, or unwanted sequestration leading to inactivation, or nonspecific binding in biological environments. Moreover, ferritin nanozymes may avoid nanozymes from forming unwanted protein corona in circulation *in vivo*.

4) Reduce toxicity and immune response caused by nanozymes *in vivo.* Using ferritin derived from human species to synthesized nanozymes may significantly reduce the immune response and toxicity caused by nanozymes during *in vivo* biomedical applications. Ferritin derived from human body does not contain any potential toxic elements that would activate inflammatory or immunological response. Nanozymes may avoid from immune response when shelled by ferritin nanocages. In addition, when nanocaged by ferritin, some toxic nanozymes, like noble metal based nanozymes, may be avoid of the contact with the main organs and thus reduce its toxicity.

5) Selectivity of ferritin nanozymes for substrates. The low substrate specificity of nanozymes is a major barrier between nanozyme and natural enzyme. Creating ferritin nanozymes by adding specific substrate or microenvironment targeting ability to ferritin nanocages may make up this barrier.

There are still challenges for ferritin nanozymes in biomedical applications.

1) Limited types of nanozymes can be synthesized in ferritin nanocages. Learning the way of biomineralization process of ferritin, Fe_3_O_4_ and Co_3_O_4_ nanozymes were synthesized in the cavity of ferritin. Exploring to load other kinds of metal oxide nanoparticles, such as Mn_3_O_4_, CuO, etc, and exhibiting different kinds of enzyme-like activities may further expand the biomedical applications of ferritin nanozymes.

2) Robust strategies to control the uniformity and size of ferritin nanozymes are needed. Controlling the amounts of metal ions and the adding rate of oxidants or reductants in the reaction system may efficiently control the uniformity and the size of ferritin nanozymes. However, the operating technique is still immature; a robust ferritin nanozyme synthesis system needs to be established.

3) Limited applications of ferritin nanozymes. Ferritin nanozymes have been used for biosensing, antibacterial and disease theranostics. However, recent reported ferritin nanozymes are seldom used for *in vivo* applications, especially for therapy applications. How to better combine the superiority of ferritin and nanozymes to create new ferritin nanozymes for effective theranostics needs further exploration.

In addition, ferritin nanozymes also inspire us to use other natural nanostructures (*e.g.* magnetosome, viral capsid) to create functional nanozymes for diseases targeted theranostics. Importantly, ferritin nanozymes provide a way for us to learn from nature to optimize and design nanozymes, thus solving problems in the field of nanozyme and nanobiology.

Taken together, ferritin nanozyme is a promising agent for disease-targeted theranostics. How to better control the size of nanozymes inside the ferritin protein cage and synthesize nanozymes with high activity and selectivity still need further exploration. Taking advantage of the BBB traversing ability of ferritin and ROS scavenging ability of nanozymes, ferritin nanozymes are expected to be used for brain disorders therapy (*e.g.* stroke, Parkinson's disease). Combining the characteristics of the tumor microenvironment (*e.g.* acidic pH, hypoxia, tumor stem cells) with the modification of ferritin nanocage may help us to create novel types of ferritin nanozymes for more effective tumor therapy.

## Figures and Tables

**Table 1:** Summary of inorganic metal-based nanomaterials synthesized in ferritin.

Synthetic Methods	Mineral Core	Ferritin Type	Core Size	Reference
Oxidation	Fe_3_O_4_	Recombinant Human HFn	4.7 ± 0.8 nm	12
			5.6 ± 0.9 nm	44
		Horse Spleen Apoferritin	5.9 ± 1.3 nm	45
	MnOOH	Horse Spleen Apoferritin	7.15 - 7.80 nm	46
		Recombinant Human HFn		
		Recombinant Human LFn		
		Horse Spleen Apoferritin	6.7 ± 1.7 nm	46
	In_2_O_3_	Horse Spleen Apoferritin	6.6 ± 0.5 nm	48
	TiO_2_	Horse Spleen Apoferritin	5.7 ± 1 nm	49
	EuOOH	Horse Spleen Apoferritin	5.7 ± 1 nm	
	Co_3_O_4_	Horse Spleen Apoferritin	6 nm	50
		Recombinant Horse liver LFn	6 nm	
		pfFn	5.7 ± 1.0 nm	16
	Ni(OH)_3_	Horse Spleen Apoferritin	7 nm	51
		Recombinant Horse liver LFn		
	Cr(OH)_3_	Horse Spleen Apoferritin	7 nm	
		Recombinant Horse liver LFn		
	FeS	Horse Spleen Apoferritin	7.8 nm	52
	CdS	Horse Spleen Apoferritin	4.0 ± 1.2 nm	53
	PbS	Horse Spleen Apoferritin	5 ± 2 nm	54
	ZnSe	Horse Spleen Apoferritin	3 nm	55
	FePO_4_	Horse Spleen Apoferritin	8 nm	56
Reduction	Au	Horse Spleen Apoferritin	6.3 ± 0.8 nm	57
	Ag	Horse Spleen Apoferritin	4.0 ± 0.5 nm	58
	Cu	Horse Spleen Apoferritin	3.0 ± 0.5 nm	58
	Pd	Horse Spleen Apoferritin	2.0 ± 0.3 nm	59
	Co	Horse Spleen Apoferritin	3.0 ± 0.5 nm	58
			3.5 nm	60
	Ni	Horse Spleen Apoferritin	3.5 ± 0.5 nm	58
			3 nm	60
	Pt	Pig Spleen Apoferritin	4.3 ± 0.9 nm	61
	CoPt	Horse Spleen Apoferritin	4.1 nm	62
	AuPd	Recombinant Horse liver LFn	2.4 ± 0.3 nm	63
Phosphatization	Silver Phosphate NPs	Horse Spleen Apoferritin	-	64

**Table 2:** Summary of ferritin nanozymes.

Ferritin Type	Nanozyme Core	Enzyme-like activity type	Nanozyme Core Size	Synthetic Methods	Applications	Reference
Recombinant Human LFn	Pt NPs	Ferroxidase	-	Reduction	Regulates iron homeostasis	65
Horse Spleen Apoferritin	Au NPs	Enhanced Ferroxidase Activity	3.03 ± 0.57 nm 4.21 ± 0.93 nm 4.84 ± 1.14 nm 3.34 ± 0.56 nm 2.81 ± 0.49 nm	Reduction	Improve *in vivo* iron uptake	66
	Ag NPs		2.37 ± 0.29 nm 3.70 ± 0.56 nm 5.92 ± 0.94 nm 5.69 ± 1.08 nm 2.71 ± 0.47 nm			
	Pt NPs		2.34 ± 0.33 nm 3.24 ± 0.56 nm 4.69 ± 0.84 nm 5.32 ± 1.06 nm 5.79 ± 1.04 nm			67
Horse Spleen Apoferritin	Fe_3_O_4_/γ-Fe_2_O_3_	Peroxidase	3.21 nm 6.83 nm	Oxidation	Detection of H_2_O_2_	68
Recombinant Human HFn	Fe_3_O_4_	Peroxidase	4.7 ± 0.8 nm	Oxidation	Tumor diagnosis	12
			5.35 ± 0.95 nm			69
			4.3 nm		Cardiovascular diseases diagnosis	17
Recombinant Human HFn	Co_X_Fe_3-X_O_4_	Peroxidase	5.53 ± 0.94 nm 5.54 ± 1.08 nm 5.81 ± 0.92 nm	Oxidation	Tumor diagnosis	69
pfFn	Co_3_O_4_	Peroxidase	5.7 ± 1.0 nm	Oxidation	Tumor diagnosis	16
Horse Spleen Ferritin	Prussian blue NPs	Peroxidase	-	Oxidation/ Deposition	Glucose Detection	70
Horse Spleen Apoferritin	Au clusters	Peroxidase	2 nm	Reduction	Glucose Detection	71
Horse Spleen Apoferritin	Fe atoms	Peroxidase	16 Fe atoms	Reduction	Glucose, Cholesterol, and Alcohol Detection	72
Horse Spleen Apoferritin	Fe-Pt NPs Fe-Pd NPs Fe-Rh NPs	Peroxidase	6 nm	Two-step Reduction		
pfFn	Ag NPs	Peroxidase	4.7 ± 1.59 nm	Reduction on the outer surface	Discoloration of nitrophenol and diazo dyes	73
	Pd NPs		4.21 ± 1.39 nm			
pfFn	Pd Nanoclusters	Oxidase	5 ± 1 nm	Reduction	Catalyze the aerobic oxidation of alcohols	74
Horse Spleen Apoferritin	Nano-CeO_2_	SOD	4.5 nm	pH dependent Disassembly-Reassembly	Scavenging of intracellular ROS	75
Horse Spleen Apoferritin	Pt NP	Catalase Peroxidase	1.87 ± 0.40 nm	Reduction	pH, temperature dependent activity	76
Horse Spleen Apoferritin	Pt NPs	Catalase SOD	2 nm	Reduction Reduction	Scavenging of intracellular ROS	77
	Pt-Au alloy NPs		3 nm			78
Horse Spleen Apoferritin	Au-Ag alloy NPs	SOD Catalase Peroxidase	-	Reduction	Cytoprotecting	79
Horse Spleen Apoferritin	Au-Ag alloy NPs	Catalyze the reduction of 4-nitrophenol	6.3 ± 1.0 nm 6.1 ± 0.7 nm 6.0 ± 0.8 nm 5.8 ± 0.8 nm 5.6 ± 0.6 nm	Reduction	Reduction of 4-nitrophenol	80
Recombinant Human HFn	Nitrogen-doped Porous Carbon Nanospheres (N-PCNSs)	Oxidase Peroxidase Catalase SOD	100 ± 10 nm	Chemical Coupling	Tumor therapy	81
Horse Spleen Apoferritin	[Cp*Ir(biot-p-L)Cl]·Sav	Artificial Transfer Hydrogenase	4.5 nm · 5.5 nm · 5.1 nm	pH dependent Disassembly-Reassembly	Reduction of cyclic imines	82
Recombinant horse LFn	[Rh(nbd)Cl]_2_	Artificial metalloenzyme	-	Inner cavity binding site immobilization	Polymerization of phenylacetylene	83

## Abbreviations

HSAF: Horse spleen apoferritin
HFn: Recombinant H-subunit ferritin
LFn: Recombinant L-subunit ferritin
pfFn: *Pyrococcus furiosus* ferritin

## References

[1] Gao L, Zhuang J, Nie L, Zhang J, Zhang Y, Gu N (2007). Intrinsic peroxidase-like activity of ferromagnetic nanoparticles. Nat Nanotechnol.

[2] Liang M, Yan X (2019). Nanozymes: from new concepts, mechanisms, and standards to applications.

[3] Gao L, Fan K, Yan X (2017). Iron oxide nanozyme: a multifunctional enzyme mimetic for biomedical applications. Theranostics.

[4] Jiang D, Ni D, Rosenkrans ZT, Huang P, Yan X, Cai W (2019). Nanozyme: new horizons for responsive biomedical applications. Chem Soc Rev.

[5] Wu J, Wang X, Wang Q, Lou Z, Li S, Zhu Y (2019). Nanomaterials with enzyme-like characteristics (nanozymes): next-generation artificial enzymes (II). Chem Soc Rev.

[6] Gao L, Yan X (2016). Nanozymes: an emerging field bridging nanotechnology and biology. Sci China Life Sci.

[7] Duan D, Fan K, Zhang D, Tan S, Liang M, Liu Y (2015). Nanozyme-strip for rapid local diagnosis of Ebola. Biosens Bioelectron.

[8] Komkova MA, Karyakina EE, Karyakin AA (2018). Catalytically synthesized prussian blue nanoparticles defeating natural enzyme peroxidase. J Am Chem Soc.

[9] Liang M, Fan K, Zhou M, Duan D, Zheng J, Yang D (2014). H-ferritin-nanocaged doxorubicin nanoparticles specifically target and kill tumors with a single-dose injection. Proc Natl Acad Sci U S A.

[10] Truffi M, Fiandra L, Sorrentino L, Monieri M, Corsi F, Mazzucchelli S (2016). Ferritin nanocages: A biological platform for drug delivery, imaging and theranostics in cancer. Pharmacol Res.

[11] Belletti D, Pederzoli F, Forni F, Vandelli MA, Tosi G, Ruozi B (2017). Protein cage nanostructure as drug delivery system: magnifying glass on apoferritin. Expert Opin Drug Del.

[12] Fan KL, Cao CQ, Pan YX, Lu D, Yang DL, Feng J (2012). Magnetoferritin nanoparticles for targeting and visualizing tumour tissues. Nat Nanotechnol.

[13] Bellini M, Mazzucchelli S, Galbiati E, Sommaruga S, Fiandra L, Truffi M (2014). Protein nanocages for self-triggered nuclear delivery of dna-targeted chemotherapeutics in cancer cells. J Control Release.

[14] Li L, Fang CJ, Ryan JC, Niemi EC, Lebron JA, Bjorkman PJ (2010). Binding and uptake of H-ferritin are mediated by human transferrin receptor-1. Proc Natl Acad Sci U S A.

[15] Rosager AM, Sørensen MD, Dahlrot RH, Hansen S, Schonberg DL, Rich JN (2017). Transferrin receptor-1 and ferritin heavy and light chains in astrocytic brain tumors: expression and prognostic value. PLoS One.

[16] Jiang B, Yan L, Zhang J, Zhou M, Shi G, Tian X (2019). Biomineralization synthesis of the cobalt nanozyme in SP94-Ferritin nanocages for prognostic diagnosis of hepatocellular carcinoma. ACS Appl Mater Interfaces.

[17] Wang T, He J, Duan D, Jiang B, Wang P, Fan K (2019). Bioengineered magnetoferritin nanozymes for pathological identification of high-risk and ruptured atherosclerotic plaques in humans. Nano Res.

[18] Laufberger V (1937). Sur la cristallisation de la ferritine. Bulletin de la Société de chimie biologique.

[19] Theil EC (1987). Ferritin - structure, gene-regulation, and cellular function in animals, plants, and microorganisms. Annu Rev Biochem.

[20] Harrison PM, Arosio P (1996). The ferritins: molecular properties, iron storage function and cellular regulation. Biochimica Et Biophysica Acta.

[21] Ford GC, Harrison PM, Rice DW, Smith JMA, Treffry A, White JL (1984). Ferritin - design and formation of an iron-storage molecule. Philos T Roy Soc B.

[22] Harrison PM (1986). The structure and function of ferritin. Biochem Educ.

[23] Santambrogio P, Levi S, Arosio P, Palagi L, Vecchio G, Lawson DM (1992). Evidence that a salt bridge in the light chain contributes to the physical stability difference between heavy and light human ferritins. J Biol Chem.

[24] Santambrogio P, Pinto P, Levi S, Cozzi A, Rovida E, Albertini A (1997). Effects of modifications near the 2-, 3- and 4-fold symmetry axes on human ferritin renaturation. Biochem J.

[25] Kang S, Oltrogge LM, Broomell CC, Liepold LO, Prevelige PE, Young M (2008). Controlled assembly of bifunctional chimeric protein cages and composition analysis using noncovalent mass spectrometry. J Am Chem Soc.

[26] Levi S, Yewdall SJ, Harrison PM, Santambrogio P, Cozzi A, Rovida E (1992). Evidence that H-chains and L-chains have cooperative roles in the iron-uptake mechanism of human ferritin. Biochem J.

[27] Bryce CFA, Crichton RR (1973). The catalytic activity of horse spleen apoferritin. Biochem J.

[28] Levi S, Luzzago A, Cesareni G, Cozzi A, Franceschinelli F, Albertini A (1988). Mechanism of ferritin iron uptake - activity of the H-chain and deletion mapping of the ferro-oxidase site - a study of iron uptake and ferro-oxidase activity of human-liver, recombinant H-chain ferritins, and of 2 H-chain deletion mutants. J Biol Chem.

[29] Mehlenbacher MR, Poli M, Arosio P, Santambrogio P, Levi S, Chasteen ND (2017). Iron oxidation and core formation in recombinant heteropolymeric human ferritins. Biochemistry.

[30] David M, Amyra T, Peter J, Pauline M, Stephen J, Alessandra L (1989). Identification of the ferroxidase centre in ferritin. Febs Lett.

[31] Finazzi D, Arosio P (2014). Biology of ferritin in mammals: an update on iron storage, oxidative damage and neurodegeneration. Arch Toxicol.

[32] Pozzi C, Di Pisa F, Bernacchioni C, Ciambellotti S, Turano P, Mangani S (2015). Iron binding to human heavy-chain ferritin. Acta Crystallogr D Biol Crystallogr.

[33] Vanessa J, Paolo A, Treffry A, Harrison PM, Mann S (1991). Influence of site-directed modifications on the formation of iron cores in ferritin. J Mol Biol.

[34] Honarmand EK, Hagedoorn PL, Hagen WR (2015). Unity in the biochemistry of the iron-storage proteins ferritin and bacterioferritin. Chem Rev.

[35] Chasteen ND, Harrison PM (1999). Mineralization in ferritin an efficient means of iron storage. J Struct Biol.

[36] Yang X, Chen-Barrett Y, Arosio P, Chasteen ND (1998). Reaction paths of iron oxidation and hydrolysis in horse spleen and recombinant human ferritins. Biochemistry.

[37] Arapova GS, Eryomin AN, Metelitza DI (1997). Role of the apoprotein in the catalytic peroxidase-like function of ferritin. Biochemistry (Mosc).

[38] Arapova GS, Eryomin AN, Metelitza DI (1998). Peroxidase activity of ferritin in aerosol OT reversed micelles in heptane. Biochemistry (Mosc).

[39] Eremin AN, Arapova GS, Metelitsa DI (1999). The role of the extent of hydration of reversed micelles of surfactants in the regulation of the peroxidase activity of ferritin and immunocomplexes of cortisol-peroxidase conjugates. Bioorg Khim.

[40] Borelli V, Trevisan E, Vita F, Bottin C, Melato M, Rizzardi C (2012). Peroxidase-like activity of ferruginous bodies isolated by exploiting their magnetic property. J Toxicol Environ Health A.

[41] Tang Z, Wu H, Zhang Y, Li Z, Lin Y (2011). Enzyme-mimic activity of ferric nano-core residing in ferritin and its biosensing applications. Anal Chem.

[42] Abdi Z, Bagheri R, Song Z, Najafpour MM (2019). Water oxidation by ferritin: a semi-natural electrode. Sci Rep.

[43] Keyes JD, Hilton RJ, Farrer J, Watt RK (2010). Ferritin as a photocatalyst and scaffold for gold nanoparticle synthesis. J Nanopart Res.

[44] Uchida M, Flenniken ML, Allen M, Willits DA, Crowley BE, Brumfield S (2006). Targeting of cancer cells with ferrimagnetic ferritin cage nanoparticles. J Am Chem Soc.

[45] Meldrum FC, Heywood BR, Mann S (1992). Magnetoferritin: *in vitro* synthesis of a novel magnetic protein. Science.

[46] Meldrum FC, Douglas T, Levi S, Arosio P, Mann S (1995). Reconstitution of manganese oxide cores in horse spleen and recombinant ferritins. J Inorg Biochem.

[47] Olsen C, Smith T, Embley J, Maxfield J, Hansen K, Peterson J (2017). Permanganate-based synthesis of manganese oxide nanoparticles in ferritin. nanotechnology.

[48] Okuda M, Kobayashi Y, Suzuki K, Sonoda K, Kondoh T, Wagawa A (2005). Self-organized inorganic nanoparticle arrays on protein lattices. Nano Lett.

[49] Klem MT, Mosolf J, Young M, Douglas T (2008). Photochemical mineralization of europium, titanium, and iron oxyhydroxide nanoparticles in the ferritin protein cage. Inorg Chem.

[50] Tsukamoto R, Iwahor K, Muraoka M, Yamashita I (2005). Synthesis of Co_3_O_4_ nanoparticles using the cage-shaped protein, apoferritin. B Chem Soc Jpn.

[51] Okuda M, Iwahori K, Yamashita I, Yoshimura H (2003). Fabrication of nickel and chromium nanoparticles using the protein cage of apoferritin. Biotechnol Bioeng.

[52] Mann S, Meldrum FC (1991). Controlled synthesis of inorganic materials using supramolecular assemblies. Adv Mater.

[53] Wong KKW, Mann S (1996). Biomimetic synthesis of cadmium sulfide-ferritin nanocomposites. Adv Mater.

[54] Turyanska L, Bradshaw TD, Li M, Bardelang P, Drewe WC, Fay MW (2012). The differential effect of apoferritin-PbS nanocomposites on cell cycle progression in normal and cancerous cells. J Mater Chem.

[55] Iwahori K, Yoshizawa K, Muraoka M, Yamashita I (2005). Fabrication of ZnSe nanoparticles in the apoferritin cavity by designing a slow chemical reaction system. Inorg Chem.

[56] Polanams J, Ray AD, Watt RK (2005). Nanophase iron phosphate, iron arsenate, iron vanadate, and iron molybdate minerals synthesized within the protein cage of ferritin. Inorg Chem.

[57] Fan RL, Chew SW, Cheong VV, Orner BP (2010). Fabrication of gold nanoparticles inside unmodified horse spleen apoferritin. Small.

[58] Galvez N, Fernandez B, Valero E, Sanchez P, Cuesta R, Dominguez-Vera JM (2008). Apoferritin as a nanoreactor for preparing metallic nanoparticles. Cr Chim.

[59] Ueno T, Suzuki M, Goto T, Matsumoto T, Nagayama K, Watanabe Y (2004). Size-selective olefin hydrogenation by a Pd nanocluster provided in an apo-ferritin cage. Angew Chem Int Edit.

[60] Galvez N, Sanchez P, Dominguez-Vera JM, Soriano-Portillo A, Clemente-Leon M, Coronado E (2006). Apoferritin-encapsulated Ni and Co superparamagnetic nanoparticles. J Mater Chem.

[61] Liu XY, Wei W, Wang CL, Yue H, Ma D, Zhu C (2011). Apoferritin-camouflaged Pt nanoparticles: surface effects on cellular uptake and cytotoxicity. J Mater Chem.

[62] Warne B, Kasyutich OI, Mayes EL, Wiggins JAL, Wong KKW (2000). Self assembled nanoparticulate Co: Pt for data storage applications. Ieee T Magn.

[63] Suzuki M, Abe M, Ueno T, Abe S, Goto T, Toda Y (2009). Preparation and catalytic reaction of Au/Pd bimetallic nanoparticles in Apo-ferritin.

[64] Dospivova D, Hynek D, Kopel P, Bezdekova A, Sochor J, Krizkova S (2012). Electrochemical behaviour of apoferritin encapsulating of silver(I) ions and its application for treatment of staphylococcus aureus. Int J Electrochem Sci.

[65] Li L, Zhang L, Carmona U, Knez M (2014). Semi-artificial and bioactive ferroxidase with nanoparticles as the active sites. Chem Commun.

[66] Sennuga A, van Marwijk J, Whiteley CG (2013). Multiple fold increase in activity of ferroxidase-apoferritin complex by silver and gold nanoparticles. Nanomed Nanotechnol.

[67] Sennuga A, van Marwijk J, Whiteley CG (2012). Ferroxidase activity of apoferritin is increased in the presence of platinum nanoparticles. Nanotechnology.

[68] Melnikova L, Pospiskova K, Mitroova Z, Kopcansky P, Safarik I (2013). Peroxidase-like activity of magnetoferritin. Microchimica Acta.

[69] Zhang T, Cao C, Tang X, Cai Y, Yang C, Pan Y (2017). Enhanced peroxidase activity and tumour tissue visualization by cobalt-doped magnetoferritin nanoparticles. Nanotechnology.

[70] Zhang W, Zhang Y, Chen Y, Li S, Gu N, Hu S (2013). Prussian Blue Modified Ferritin as Peroxidase Mimetics and Its Applications in Biological Detection. J Nanosci Nanotechnol.

[71] Jiang X, Sun C, Guo Y, Nie G, Xu L (2015). Peroxidase-like activity of apoferritin paired gold clusters for glucose detection. Biosens Bioelectron.

[72] Zhang W, Liu X, Walsh D, Yao S, Kou Y, Ma D (2012). Caged-protein-confined bimetallic structural assemblies with mimetic peroxidase activity. Small.

[73] Peskova M, Ilkovics L, Hynek D, Dostalova S, Sanchez-Carnerero EM, Remes M (2019). Detergent-modified catalytic and enzymomimetic activity of silver and palladium nanoparticles biotemplated by Pyrococcus furiosus ferritin. J Colloid Interf Sci.

[74] Kanbak-Aksu S, Nahid Hasan M, Hagen WR, Hollmann F, Sordi D, Sheldon RA (2012). Ferritin-supported palladium nanoclusters: selective catalysts for aerobic oxidations in water. Chem Commun.

[75] Liu X, Wei W, Yuan Q, Zhang X, Li N, Du Y (2012). Apoferritin-CeO_2_ nano-truffle that has excellent artificial redox enzyme activity. Chem Commun.

[76] Fan J, Yin JJ, Ning B, Wu X, Hu Y, Ferrari M (2011). Direct evidence for catalase and peroxidase activities of ferritin-platinum nanoparticles. Biomaterials.

[77] Zhang L, Laug L, Munchgesang W, Pippel E, Gosele U, Brandsch M (2010). Reducing stress on cells with apoferritin-encapsulated platinum nanoparticles. Nano lett.

[78] Zhang L, Fischer W, Pippel E, Hause G, Brandsch M, Knez M (2011). Receptor-mediated cellular uptake of nanoparticles: a switchable delivery system. Small.

[79] Dashtestani F, Ghourchian H, Najafi A (2019). Silver-gold-apoferritin nanozyme for suppressing oxidative stress during cryopreservation. Mater Sci Eng C Mater Biol Appl.

[80] Shin Y, Dohnalkova A, Lin Y (2010). Preparation of homogeneous gold-silver alloy nanoparticles using the apoferritin cavity. J Phys Chem C.

[81] Fan K, Xi J, Fan L, Wang P, Zhu C, Tang Y (2018). *In vivo* guiding nitrogen-doped carbon nanozyme for tumor catalytic therapy. Nat Commun.

[82] Hestericova M, Heinisch T, Lenz M, Ward TR (2018). Ferritin encapsulation of artificial metalloenzymes: engineering a tertiary coordination sphere for an artificial transfer hydrogenase. Dalton Trans.

[83] Ke Z, Abe S, Ueno T, Morokuma K (2012). Catalytic mechanism in artificial metalloenzyme: QM/MM study of phenylacetylene polymerization by rhodium complex encapsulated in apo-Ferritin. J Am Chem Soc.

[84] He J, Fan K, Yan X (2019). Ferritin drug carrier (FDC) for tumor targeting therapy. J Control Release.

[85] Jiang B, Zhang R, Zhang J, Hou Y, Chen X, Zhou M (2019). GRP78-targeted ferritin nanocaged ultra-high dose of doxorubicin for hepatocellular carcinoma therapy. Theranostics.

[86] Yang Z, Wang XY, Diao HJ, Zhang JF, Li HY, Sun HZ (2007). Encapsulation of platinum anticancer drugs by apoferritin.

[87] Pandolfi L, Bellini M, Vanna R, Morasso C, Zago A, Carcano S (2017). H-ferritin enriches the curcumin uptake and improves the therapeutic efficacy in triple negative breast cancer cells.

[88] Kuruppu AI, Zhang L, Collins H, Turyanska L, Thomas NR, Bradshaw TD (2015). An Apoferritin-based drug delivery system for the tyrosine kinase inhibitor gefitinib. Adv Healthc Mater.

[89] Li L, Munoz-Culla M, Carmona U, Lopez MP, Yang F, Trigueros C (2016). Ferritin-mediated siRNA delivery and gene silencing in human tumor and primary cells. Biomaterials.

[90] Fan K, Zhou M, Yan X (2017). Questions about horse spleen ferritin crossing the blood brain barrier via mouse transferrin receptor 1. Protein Cell.

[91] Mendes-Jorge L, Ramos D, Valença A, López-Luppo M, Pires VMR, Catita J (2014). L-ferritin binding to scara5: a new iron traffic pathway potentially implicated in retinopathy. PLoS One.

[92] Conti L, Lanzardo S, Ruiu R, Cadenazzi M, Cavallo F, Aime S (2016). L-ferritin targets breast cancer stem cells and delivers therapeutic and imaging agents. Oncotarget.

[93] Li JY, Paragas N, Ned RM, Qiu A, Viltard M, Leete T (2009). Scara5 is a ferritin receptor mediating non-transferrin iron delivery. Dev Cell.

[94] Chen TT, Li L, Chung DH, Allen CD, Torti SV, Torti FM (2005). TIM-2 is expressed on B cells and in liver and kidney and is a receptor for H-ferritin endocytosis. J Exp Med.

[95] Todorich B, Zhang X, Slagle-Webb B, Seaman WE, Connor JR (2008). Tim-2 is the receptor for H-ferritin on oligodendrocytes. J Neurochem.

[96] Han J, Seaman WE, Di X, Wang W, Willingham M, Torti FM (2011). Iron uptake mediated by binding of H-ferritin to the TIM-2 receptor in mouse cells. PLoS One.

[97] Fan K, Jia X, Zhou M, Wang K, Conde J, He J (2018). Ferritin nanocarrier traverses the blood brain barrier and kills glioma. ACS Nano.

[98] Chiou B, Lucassen E, Sather M, Kallianpur A, Connor J (2018). Semaphorin4A and H-ferritin utilize Tim-1 on human oligodendrocytes: a novel neuro-immune axis. Glia.

[99] Sevcenco AM, Paravidino M, Vrouwenvelder JS, Wolterbeek HT, van Loosdrecht MC, Hagen WR (2015). Phosphate and arsenate removal efficiency by thermostable ferritin enzyme from Pyrococcus furiosus using radioisotopes. Water Res.

[100] Fisher J, Devraj K, Ingram J, Slagle-Webb B, Madhankumar AB, Liu X (2007). Ferritin: a novel mechanism for delivery of iron to the brain and other organs. Am J Physiol Cell Physiol.

[101] Zhen Z, Tang W, Guo C, Chen H, Lin X, Liu G (2013). Ferritin nanocages to encapsulate and deliver photosensitizers for efficient photodynamic therapy against cancer. ACS Nano.

[102] Chen ZW, Yin JJ, Zhou YT, Zhang Y, Song L, Song MJ (2012). Dual enzyme-like activities of iron oxide nanoparticles and their implication for diminishing cytotoxicity. ACS Nano.

